# Direct effects of transcranial electric stimulation on brain circuits in rats and humans

**DOI:** 10.1038/s41467-018-02928-3

**Published:** 2018-02-02

**Authors:** Mihály Vöröslakos, Yuichi Takeuchi, Kitti Brinyiczki, Tamás Zombori, Azahara Oliva, Antonio Fernández-Ruiz, Gábor Kozák, Zsigmond Tamás Kincses, Béla Iványi, György Buzsáki, Antal Berényi

**Affiliations:** 1 MTA-SZTE “Momentum” Oscillatory Neuronal Networks Research Group, Department of Physiology, University of Szeged, Szeged, 6720 Hungary; 2 The Neuroscience Institute, New York University, New York, NY 10016 USA; 3 Department of Pathology, University of Szeged, Szeged, 6725 Hungary; 4 Department of Neurology, University of Szeged, Szeged, 6725 Hungary; 5 Department of Neurology, New York University, New York, NY 10016 USA; 6 Center for Neural Science, New York University, New York, NY 10016 USA

## Abstract

Transcranial electric stimulation is a non-invasive tool that can influence brain activity; however, the parameters necessary to affect local circuits in vivo remain to be explored. Here, we report that in rodents and human cadaver brains, ~75% of scalp-applied currents are attenuated by soft tissue and skull. Using intracellular and extracellular recordings in rats, we find that at least 1 mV/mm voltage gradient is necessary to affect neuronal spiking and subthreshold currents. We designed an ‘intersectional short pulse’ stimulation method to inject sufficiently high current intensities into the brain, while keeping the charge density and sensation on the scalp surface relatively low. We verify the regional specificity of this novel method in rodents; in humans, we demonstrate how it affects the amplitude of simultaneously recorded EEG alpha waves. Our combined results establish that neuronal circuits are instantaneously affected by intensity currents that are higher than those used in conventional protocols.

## Abstract (web summary)

Though transcranial electric stimulation has been used to influence brain activity, it is debated whether neuronal spiking activity is directly affected by commonly-used protocols. Here, the authors quantify the voltage gradients necessary to instantaneously affect neuronal spiking and show that they are higher than commonly-used protocols.

## Introduction

Electric fields, generated either by neurons themselves or applied externally, can affect the transmembrane potential of neurons and, consequently, the probability of occurrence of action potentials^1–3^. Forced electric fields, induced either locally (e.g., by deep brain stimulation) or non-invasively through the scalp (e.g., by transcranial electrical stimulation; TES)^4–7^, can probe neural patterns and potentially to ameliorate brain disease^8^. Yet, there is no accepted theory to explain how TES affects neuronal circuits in the brain, mainly because the physiological mechanisms of TES are not well understood. Electrical stimulation of the scalp can affect brain activity in multiple indirect ways, including activation of peripheral nerves^7,9,10^, retina^11^, the vestibular apparatus, astrocytes, perivascular elements^12,13^, and placebo effects^14^. Given the important role of brain oscillations in cognition, an often-stated explicit goal of TES is to bias brain rhythms acutely or chronically^15,16^, as opposed to inducing indirect peripheral effects.

For many therapeutic applications, it is desirable to affect neurons promptly (e.g., to terminate epileptic seizures), in a regionally constrained manner to reach maximum on-target effects and reduce effects on unintended brain networks^4,5^. Achieving spatially precise effects by scalp-applied currents requires knowledge about the spread of electric fields in the human head^17^ and the use of novel methods of current delivered through multiple electrodes^18,19^.

The effectiveness of currently used TES protocols on local neuronal networks is a subject of extensive debate^14–16,20,21^. At least two factors contribute to this controversy. First, the large electric fields induced by alternating current TES (transcranial alternating current stimulation; tACS) often prevents simultaneous measurement of electric (electroencephalographic, EEG), magnetic (magnetoencephalographic, MEG), or imaging (blood oxygen-level dependent, BOLD) signals^22^. Recent experiments attempted to alleviate the amplifier saturation problem and remove the stimulus artifacts^21–23^. However, in those experiments the expected brain rhythm entrainment was examined at the same frequency of the applied TES (e.g., 10 Hz tACS induced increased power in the alpha band), raising the possibility that large tACS artifacts which are several thousand-fold larger than the scalp signal, or a harmonic of the artifact, have contaminated the results. A second indirect approach takes the voltage gradients shown experimentally to produce spike entrainment and estimates the corresponding current intensity applied at the scalp surface. However, translation of results obtained from models^24^, in vitro observations^21,22,25,26^, and experiments performed on experimental animals^6,27^ to humans is complicated by an incomplete understanding of how skin, subcutaneous soft tissue, skull, cerebrospinal fluid, and brain folding affects current spread^28^. While strong stimulation (>50 mA; 0.5 ms pulses) delivered through intracranial screw electrodes in anesthetized patients has shown convincing brain network-induced effects^29,30^, the current intensity applied to the scalp needed to acutely affect neuronal patterns is yet to be established^5,31,32^.

The goal of the experiments presented in this paper was to identify the conditions under which neuronal spikes and local circuits could be directly affected by TES. To accomplish this goal, we first determined the voltage gradients necessary to affect the membrane potential and neuronal spiking in the intact rat brain, corresponding to approximately 1 mV/mm. Second, we introduced a novel fast pulse stimulation method that allowed simultaneous recording of electrical activity and focusing induced fields to target brain structures. We verified the validity of this method in rats and tested it in human subjects. Third, we measured the impact of scalp, soft tissue, and skull on current spread and quantified the induced fields in the brain of human cadavers, and found that only approximately 25% of the scalp-applied current enters the brain. Finally, we determined the current levels of TES necessary to affect the amplitude of alpha waves in human subjects. Our direct measurements and indirect estimation provided concordant results and established that in humans at least 4–6 mA currents should be applied by conventional tACS electrodes to reliably and instantaneously affect neuronal circuits.

## Results

### Subcutaneous and transcutaneous electric stimulation in rats

**Fig. 1:**
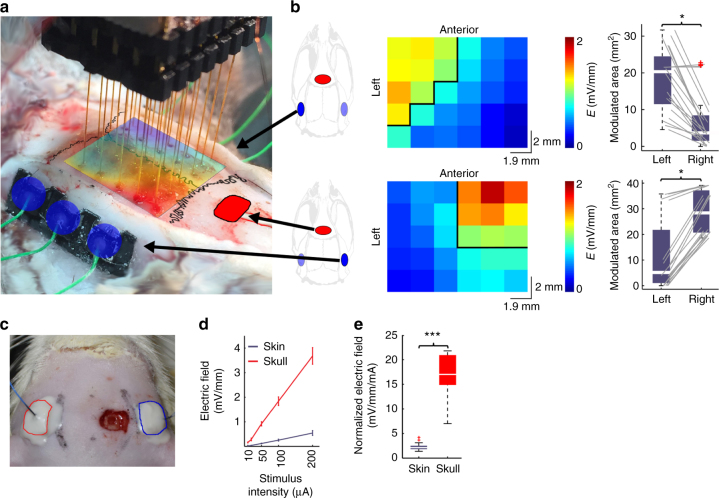
Intracerebral electric field distribution and magnitude during subcutaneous or transcutaneous stimulation. **a** Intraoperative photograph of the measurement of intracerebral electric fields by a 6-by-5 electrode matrix in an anesthetized rat. Red: cathodal, blue: anodal gel electrodes glued onto the skull surface. The spline interpolated map of the measured intracerebral gradients shown in **b** (bottom) is superimposed on the skull surface. **b** Map of the measured intracerebral gradients in the horizontal plane. The boundary where the gradients are >1 mV/mm is marked by black lines (applied intensity: 100 µA). Right, boxplots with whiskers indicate group results (full data set is superimposed in gray). Proper placement of the stimulating electrodes can restrict the extent of the effective electric field gradients to either the left (upper plot) or the right (lower plot) hemisphere (*P* = 0.04 and 0.02; *n* = 20 in 4 rats each, Mann–Whitney *U*-test). **c** Photograph of scalp stimulation electrodes and the small hole in the skull through which intracellular recordings were made. **d**, **e** Transcutaneous stimulation at the same stimulus intensities generated several-fold weaker electric fields compared to subcutaneous stimulation (*P* < 0.005, *n* = 20 in 4 rats)

Previous in vivo experiments in rodents were performed using stimulation electrodes placed on the skull^4,6^ (i.e., subcutaneous TES) and demonstrated both stimulus-locked firing of neurons in both neocortex and subcortical structures^6^ as well as effects on the amplitude of intracerebrally recorded local field potentials (LFP)^4^. Because skin and head musculature surrounding the skull represent a large shunt which can diffuse the applied current, first we examined the current loss between scalp and the brain. Using implanted arrays of electrodes and stimulation pads placed on the parietal bone and pre-bregma frontal bone, we measured the intracerebral voltage gradients in the horizontal plane (Fig. 1a, b). Subcutaneous TES at 50 µA alternating current was sufficient to induce ~1 mV/mm electric fields (Fig. 1b, d). The distribution of the electric fields could be biased by different configurations of bipolar stimulation (Fig. 1b, left-side stimulation: 20.33 (IQR = 11.49–24.52) and 3.65 (IQR = 1.58–8.39) mm^2^ exceeding 1 mV/mm on the left and right hemisphere, respectively; *P* = 0.04; right-side stimulation: 5.16 (IQR = 1.01–21.76) and 28.37 (IQR = 20.72–31.18) mm^2^ exceeding 1 mV/mm on the left and right hemisphere, respectively; *P* = 0.02; Mann–Whitney *U*-test and *n* = 20 × 2 in both cases). In contrast, applying the current to the shaved scalp (transcutaneous TES) through the same size electrodes resulted in an 80 ± 5% current loss, independent of the stimulus intensity (Fig. 1c–e; subcutaneous stimulation: 17.01 (IQR = 14.96–20.85) mV/mm/mA; transcutaneous stimulation: 2.14 (IQR = 1.9–2.44) mV/mm/mA; *P* < 0.001; paired *t*-test; *n* = 20 × 2).

**Fig. 2:**
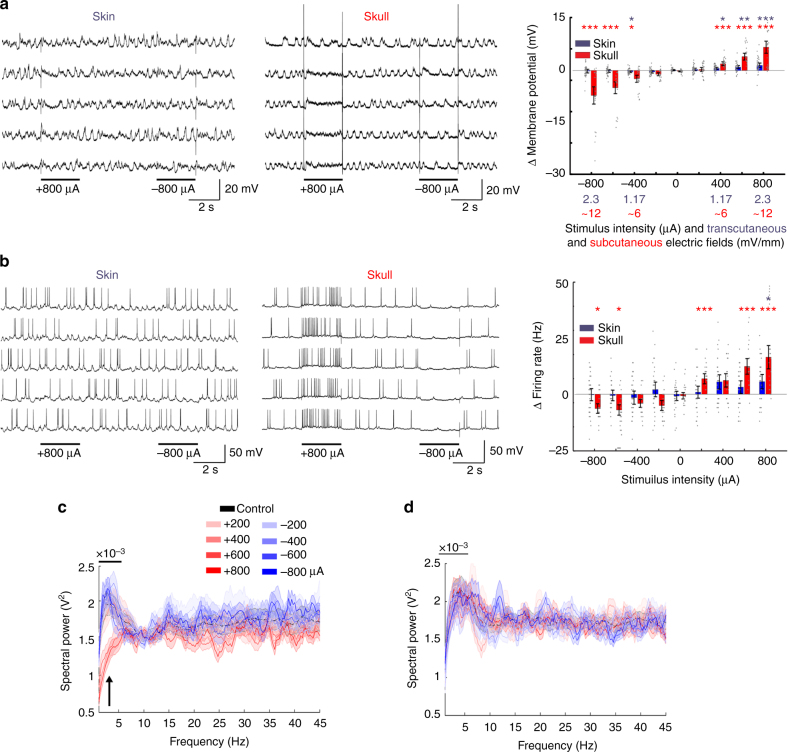
Modulating neuronal activity by subcutaneous or transcutaneous stimulation. **a** Subthreshold membrane potential changes of cortical neurons by transcutaneous and subcutaneous direct current stimuli. *V*_m_ was held below spiking by intracellularly injected hyperpolarizing current. Five representative trials are shown for each arrangement. Right panel, group effects (*n* = 40 trials from 8 neurons of 3 rats for transcutaneous and *n* = 25 trials from 5 neurons of 4 rats for subcutaneous experiments). Note linear changes of *V*_m_ with changing polarity and amplitude of forced fields (*R* = 0.86, *P* < 0.005 for transcutaneous and *R* = 0.97, *P* < 0.005 for subcutaneous stimulation, *n* = 13 trials, each; asterisks mark significant differences against control condition, *n* = 25/40 for subcutaneous/transcutaneous trials). For each stimulus intensity, the generated electric field strengths are shown at the bottom of the plot in blue and red for transcutaneous and subcutaneous stimuli, respectively. **b** Same as **a** but for affected spiking frequency by applied fields (*R* = 0.80, *P* = 0.007 for transcutaneous and *R* = 0.95, *P* < 0.005 for subcutaneous stimulation, *n* = 13, each; asterisks mark significant pairwise differences against control condition, *n* = 25/35 for subcutaneous/transcutaneous trials). **c**, **d** Changes of *V*_m_ power spectra in response to subcutaneous (**c**, *n* = 30 trials) and transcutaneous (**d**, *n* = 35 trials) stimuli. Note the lack of a significant effect with transcutaneous stimulation and prominent decrease of delta power (1–5.4 Hz) at +600 and +800 µA conditions compared to control (arrow; *P* < 0.005, *n* = 30 power value pairs at each frequency bin from 6 animals; Mann–Whitney *U*-test with Bonferroni correction)

In a more direct physiological comparison, we tested the effects of externally applied direct currents on the intracellularly recorded transmembrane potential (*V*_m_) and spiking of neurons in the deep layers of the visual cortex (Fig. 2a–d). Subcutaneous (skull) stimulation exerted clear and predictable effects on *V*_m_. Depending on the polarity of the stimulation, *V*_m_ became depolarized or hyperpolarized in a relatively linear manner (Fig. 2a; Pearson’s linear correlation, *R* = 0.86, *P* = 0.002 for transcutaneous and *R* = 0.97, *P* < 0.001 for subcutaneous stimulation, *n* = 13, each), and decreased or increased the number of action potentials, respectively (Fig. 2b; Pearson’s linear correlation, *R* = 0.80, *P* = 0.007 for transcutaneous and *R* = 0.95, *P* < 0.001 for subcutaneous stimulation, *n* = 13, each). Subcutaneous depolarizing pulses significantly decreased *V*_m_ (paired *t*-test with Bonferroni correction; *P* <0.001, <0.001, <0.001 for 400, 600, 800 µA vs. 0 µA; *n* = 25 membrane potential difference values), increased firing rate (paired *t*-test with Bonferroni correction; *P* = 0.001, <0.001, <0.001 for 200, 600, 800 µA vs. 0 µA; *n* = 25 firing rate difference values) and reduced *V*_m_ power in delta frequency band (1–5 Hz; +600 to +800 µA, Mann–Whitney *U*-test with Bonferroni correction; *P* < 0.005, *n* = 30 power value pairs at each frequency bin), indicating that subcutaneous stimulation affected many other neurons as well (Fig. 2c). Hyperpolarizing pulses exerted opposite effect with similar magnitudes on *V*_m_ (paired *t*-test with Bonferroni correction; *P = *0.003, 0.004 and 0.046 for −800, −600, −400 µA vs. 0 µA; *n* = 25 membrane potential difference values) and reduced firing rate (paired *t*-test with Bonferroni correction; *P* = 0.044, 0.028 for −800 and −600 µA vs. 0 µA; *n* = 25 firing rate difference values). Using the same current intensities, transcutaneous (scalp) stimulation produced much smaller and more variable effects (*V*_m_ was affected at anodal 400–800 µA but not by cathodal pulses; paired *t*-test with Bonferroni correction; *P* = 0.044, 0.008, and 0.003 for 400, 600, and 800 µA vs. 0 µA; *n* = 40 membrane potential difference values, and even the highest current intensities failed to affect delta power *V*_m_ or higher frequencies; Fig. 2d; Mann–Whitney *U*-test with Bonferroni correction; *P* > 0.05; *n* = 35 × 150 spectral amplitude values for all conditions). Spiking activity by transcutanenous stimulation was affected at only 800 µA depolarizing pulses (paired *t*-test with Bonferroni correction; *P* = 0.046 for 800 µA vs. 0 µA; *n* = 35 firing rate difference values), corresponding to intracranial fields of approximately 2 mV/mm (Fig. 2b, d). In summary, electric fields applied either subcutaneously or transcutaneously, which induce at least 1 mV/mm intracerebral voltage gradient, can affect spiking activity, but stronger fields are needed to affect network oscillations.

### Focused TES effect by Intersectional Short Pulse stimulation

**Fig. 3:**
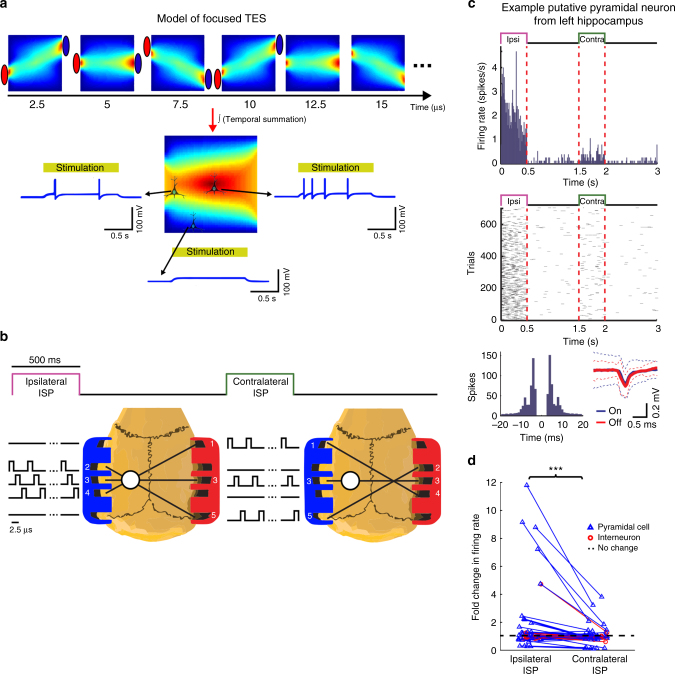
Intersectional Short Pulse (ISP) stimulation can spatially focus induced fields. **a** Leaky integrate-and-fire neuron model cartoon to demonstrate the concept of ISP stimulation. Stimulus current is delivered sequentially through three independent electrode pairs generating a continuously changing intracerebral gradient pattern. Neuronal cell membranes can integrate these patterns due to their relatively slow membrane time constant (10 ms). Consequently, neurons at the cross-section of the current flow axes integrate all three stimuli, and become more strongly entrained than neurons located outside the focus. **b** Experimental protocol to measure the efficacy of ISP. White circle marks the craniotomy for the example left hippocampal neuron shown in **c**. The contralateral craniotomy is not displayed for simplicity. 3D-printed gel electrode holders (anode = left; cathode = right) were attached to the temporal bones bilaterally with five electrodes on each side. Three electrode pairs were programmed to target the ISP beams on either the left or the right hemisphere (serving as ISP_ipsi_ and ISP_contra_ conditions for the example in **c**, respectively). Each electrode pair was pulsed for 2.5 µs and the pulses cycled through the three pairs for 500 ms followed by non-stimulated 1-s control periods. This sequence was repeated to alternatingly stimulate the right or left hemisphere. The idealized beam crossings shown here may be modified by the inhomogeneity of brain structures and ventricles. **c** Response of an example neuron. The putative pyramidal cell from the left hippocampus was strongly excited by the ipsilateral focal stimulation, as shown by peristimulus time histograms (top panels) and raster plots (middle panels). ISP stimulation did not affect isolation of single units as demonstrated by the similar autocorrelograms and identical spike waveforms during stimulation and control periods. **d** Fold-changes of normalized firing rates of the significantly modulated cells from the left (*n* = 32 units) and from the right hippocampus (*n* = 23 units) show lateralized effect of the ISP stimulation (*P* = 0.001; ISP_ipsilateral_ vs. ISP_contralateral_ Wilcoxon signed rank test)

For many experimental and clinical applications^5,22^, it would be desirable to apply TES in a spatially targeted manner and simultaneously monitor the induced electrical changes to verify online effectiveness. Because the scalp, skull, and brain conduct current in a homogenous manner, simultaneous application of TES through multiple electrode pairs cannot induce a spatially confined effect (see Supplementary Figure 6b). Our proposed solution to achieve spatially targeted TES effects is to apply spatio-temporally rotating Intersectional Short Pulse (ISP) stimulation. This method exploits the short integration time constant of the neuronal membrane (5–20 ms), a mechanism that can temporally integrate multiple electrical gradients with similar vector directions (Fig. 3a, Supplementary Figure 1)^31^. An added advantage of fast pulse stimulation (2.5 or 10 µs pulse width with 5 or 50 µs pause, depending on the number of electrode pairs) is that the transients of high frequency pulses affect simultaneously recorded LFP or neuronal spikes (1 Hz–5 kHz; 20 kHz sampling) substantially less than conventional tACS and they do not saturate recording amplifiers even at relatively high intensities.

To test our model prediction of focal effect in rats, current pulses were delivered sequentially and in a spatially asymmetric manner through independently programmable isolated current generators, which were connected to a three-dimensional (3-D) printed gel-electrode strip glued to the temporal bone surface (Fig. 3b). Unit activity was recorded bilaterally in the hippocampal CA1 region with silicon probes (7 anesthetized rats and 1 chronically implanted rat). The hemisphere target of the bipolar stimulation configuration was alternated (Fig. 3b, c; Supplementary Figure 2a). The effectiveness of the ISP stimulation on spatially targeted entrainment of single unit activity is illustrated for an example neuron from the left hippocampus (Fig. 2c). The artifacts of the short duration stimulation pulses did not affect the recording quality as demonstrated by the similar spike waveforms and spike autocorrelograms of the unit during stimulation and stimulation-free periods (Fig. 3c). Of the 127 isolated single units, 55 were significantly affected by at least one configuration of the stimulation protocol (32 increased and 23 decreased, significance threshold: *P* < 0.05; Wilcoxon signed rank test; *n* = 300 firing rate values for each neuron and condition, all tested against baseline condition). To quantify the focusing effect of ISP, we calculated the fold-change of unit discharge in the left and right hippocampus, respectively. Using only three rotating dipoles, the current-focusing effect of ISP resulted in a several-fold increase in induced unit discharge between the targeted and non-targeted hemispheres (Fig. 3d, 1.8 ± 2.35-fold vs. 1.017 ± 0.63-fold; mean ± SD; *P* = 0.001; Wilcoxon signed rank test; *n* = 55 units).

In four of the above animals (three anesthetized and one chronic; 77 units), the spatial selectivity of the ISP method was compared to traditional direct current (DC) pulses (for DC stimulation, the electrodes in the same hemisphere were short-circuited; the same current intensity was used for DC and ISP stimulation). For each protocol, 500 ms stimulation epochs alternated with 1000 ms stimulus-free epochs using the following sequence: ISP_left_, ISP_right_, DC_left_, DC_right_. Eighteen (ISP) and 10 (DC) neurons showed significant firing rate changes to at least one stimulation configuration (significance threshold; *P < *0.05; Wilcoxon signed rank test; *n* = 340 firing rate values for each neuron and condition, tested against baseline). Of the 18 ISP-driven hippocampal neurons, eight (44%) responded differentially to ISP_left_ and ISP_right_ conditions (significance threshold: *P < *0.05; paired *t*-test; *n *= 340 firing rate pairs for each neuron). Of the 10 DC-driven neurons, only one neuron (10%) showed significant difference to left (anode) vs. right (anode) stimulation (significance threshold: *P* < 0.05; paired *t*-test; *n* = 340 firing rate pairs for each neuron). In summary, the ISP stimulation affected neural activity in spatially targeted manner, even though skull thickness, brain geometry, tissue anisotropy, and ventricles likely distorted current spread.

### Measuring current spread in human cadavers

**Fig. 4:**
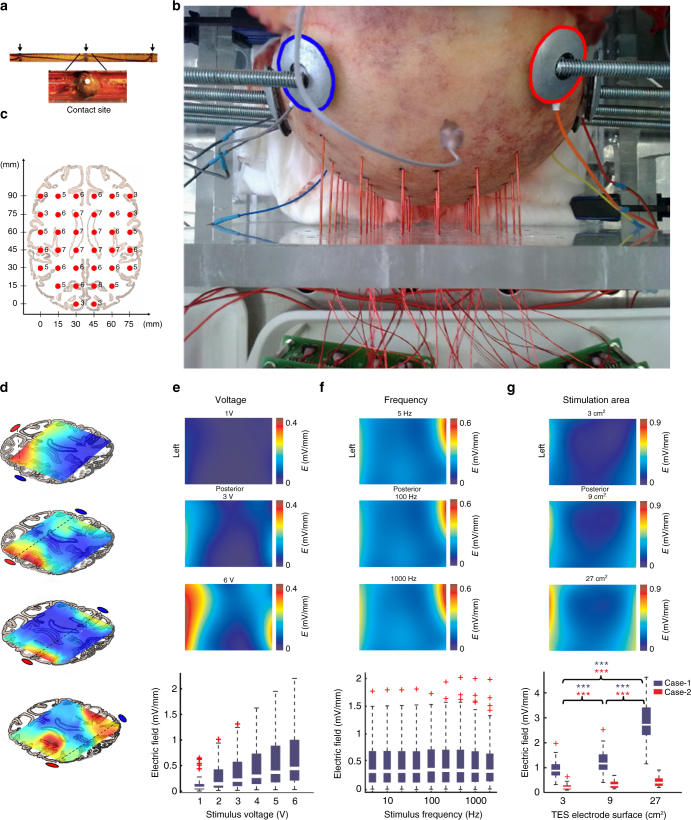
Measuring induced intracerebral electric fields in human cadavers. **a** Photomicrograph of the custom-made multicontact electrodes used in the cadaver experiments. **b** Photograph of the skull with drilled holes and inserted electrodes. A needle electrode in the sagittal plane on the forehead served as reference for the recordings. Ag/AgCl electrodes, marked by blue and red circles for negative and positive polarity, respectively, were fixed to the skull by conductive gel and secured by rubber-lined washers fixed to the plexiglass frame by threaded rods. **c** Stereotaxic coordinates of the electrode shanks. Numbers denote the number of recording sites for each electrode shank. Electrode tips (and adjacent sites) were positioned at the same depth to sample distinct horizontal planes. The depth coverage of our electrodes was 3–7 cm (depending on the number of contact sites). **d** The effect of different stimulation electrode configurations on the distribution of voltage gradients displayed on a single horizontal slice. Position of the stimulating electrodes determines the location of maximal intracranial effect. Voltage gradients were calculated the same way as in Fig. 1b. **e**–**g** Effect of stimulus intensity, frequency, and electrode size on intracerebral voltage gradients, respectively. Top three panels denote example gradient maps in the horizontal plane, bottom graphs show population data. **e** Electric field strength is a linear function of applied stimulus intensity (*R* = 0.52; *P* < 0.001; *n* = 48 gradient values in two different arrangements in 4 cadavers). **f** Stimulus frequency between 5 and 1000 Hz has a minor effect on intracerebral gradients (one-way ANOVA; *F*(8, 891) = 0.0667, *P* = 0.99, mean ± 2 SD is shown, *n* = 900 gradient values in 5 cadavers). **g** Increasing electrode size increases the magnitude of electric fields in a constant voltage mode, as the aggregate resistance decreases (*n* = 60 from 2 cadavers, *P* < 0.001 for all conditions)

Currently, the best estimates of the currents needed to induce electric fields of sufficient magnitude intracranially are offered by in silico modeling of the human head^5,33^. However, there are many uncertainties in such modeling. As an alternative to modeling, we carried out high spatial density, 3-D intracerebral measurements in cadaver brains in situ (*n* = 11; Supplementary Tables 1 and 2). Prior to each experiment, the subdural space was filled with physiological saline (0.9% NaCl) solution to replace the cerebrospinal fluid lost during the insertion of the recording electrodes. Thirty-six custom-made multisite electrodes (three to seven sites per electrode, 198 in total, Fig. 4a, Supplementary Figure 3) were inserted into the brain through holes drilled through the calvarium after removing the soft tissue around the skull (Fig. 4b, c) to create a 3-D montage. As the overall volume of the removed skull was negligible compared to the total skull volume, and the polyimide electrode shafts were tightly sealed, the conduction/isolation properties of the skull were not affected. A needle electrode placed into the sagittal sulcus on the forehead served as the reference electrode. Four or seven pairs of Ag/AgCl stimulation electrodes were fixed to the skull surface bilaterally by conductive electrode gel (Fig. 4b, c). Using either DC pulses or alternating current stimulation (Supplementary Figure 4) showed that, similar to the rat, the highest electric fields occurred in the neocortex near the stimulation electrodes (Fig. 4d; Supplementary Figure 5). The generated gradient patterns were independent of whether voltage or current mode was used for stimulation (Supplementary Figure 6a), and the relationship between applied current or voltage and the measured electric fields was linear (Fig. 4e; Pearson’s linear correlation; *R* = 0.52; *P* < 0.001; *n* = 48). The frequency of stimulation had only a small effect on the magnitude of the induced fields (Fig. 4f; one-way ANOVA; *P* = 0.99; *F*(8, 891) = 0.06; *n* = 900 trials from 5 cadavers). These results demonstrate ohmic properties of the brain^34^, the surrounding skull, and soft tissue with negligible capacitive components^35^. When multiple electrodes were simultaneously stimulated, the common conductive soft tissue summed the independently applied dipoles, as shown by an equivalent circuit diagram (Supplementary Figure 6b). As a result, simultaneously applied multiple stimuli at different locations but with an increasing phase separation gradually decreased the magnitude of electric fields (Supplementary Figure 6c). As expected, larger size electrodes induced larger electric fields (Fig. 4g, case 1: 0.94 ± 0.041, 1.25 ± 0.05, 2.84 ± 0.097 mV/mm, *P* < 0.001 for all comparison; and case 2: 0.23 ± 0.01, 0.32 ± 0.017, 0.43 ± 0.09 mV/mm, *P* < 0.001 for all comparison; mean ± SEM; *n* = 60 gradient values)^36^. Unilateral placement of the electrodes restricted the induced electric fields largely to the selected hemisphere (Fig. 4d; Supplementary Figure 7; *E*_targeted_ = 0.9 ± 0.06 mV/mm and *E*_non-targeted_ = 0.49 ± 0.03 mV/mm, mean ± SD; *n* = 30 cortical electric field values), similar to the results obtained in rats (Fig. 1b).

**Fig. 5:**
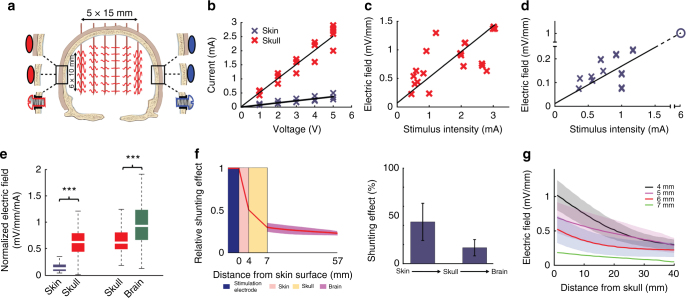
Skin and subcutaneous soft tissue diffuses scalp-applied current in cadaver brains. **a** Schematic of the experimental arrangement for transcutaneous, subcutaneous, and epidural stimulation. Example signal traces recorded in a coronal plane. Note the phase reversal of sinusoid voltage traces between the two sides. **b** Both transcutaneous and subcutaenous stimulation show intensity-independent linear (ohmic) properties (*n* = 81 in four different arrangements in 10 cadavers, *R* = 0.92, *P* < 0.001 for subcutaneous, and *n* = 14 in 6 cadavers, *R* = 0.86, *P* < 0.001 for transcutaneous stimulation; raw data and fitted line are shown), which allows the calculation of voltage–current relationship. **c**, **d** Subcutaneous stimulation (**c**, *R* = 0.56, *P* < 0.001, *n* = 29 in 10 cadavers) elicited several-fold larger intracerebral gradients compared to transcutaneous stimulation (**d**, *R* = 0.8, *P* < 0.001, *n* = 16 in 6 cadavers). Extrapolation from the measured data indicates that approximately 6 mA transcutaneous current can induce 1 mV/mm intracerebral electric field (circle). Raw data and fitted lines are shown. **e** Ratios of induced intracerebral fields and stimulus amplitude in trancutaneous vs. subcutaneous (*P* < 0.001, *n* = 36 in two different arrangements in 6 cadavers), and subcutaneous vs. epidural stimulation (*P < *0.001, *n* = 60 in 3 cadavers). **f** 58 ± 7% of the applied current is shunted by skin and soft tissue and a further 16 ± 8% is attenuated by the serial resistance of the skull. **g** Effect of skull thickness on induced fields (*n* = 64 in 8 cadavers)

Figure 5 presents comparisons between transcutaneous (scalp), subcutaneous (skull with scalp removed), and direct epidural stimulation results (Supplementary Table 3). Transcutaneous experiments used a limited set of recording electrodes to one plane of the 3-D matrix (total of 28 contact sites on four electrodes in 6 cadavers) introduced via small individual incisions of the otherwise intact scalp. The voltage–current relationship remained linear for transcutaneous stimulation as well but the slopes were strongly reduced (Fig. 5b; Pearson’s linear correlation; *R*_subcutaneous_ = 0.92 and *R*_transcutaneous_ = 0.86; *P* < 0.001 in both cases; *n* = 14 and 81 for transcutaneous and subcutaneous conditions, respectively). Comparison of the current–electric field relationships indicated that approximately 2 mA subcutaneously applied current was sufficient to induce a 1-mV/mm field maximum (Fig. 5c; Pearson’s linear correlation; *R* = 0.56; *P* < 0.001; *n* = 29). In contrast, the current vs. electric field slope was decreased three-fold when current was applied to the scalp rather than to the skull (Fig. 5c, d; *E*_subcutaneous_(mV/mm) = 0.41 × *I*(mA) + 0.15; *E*_transcutaneous_(mV/mm) = 0.13 × *I*(mA) + 0.04). Extrapolation of transcutaneous stimulation results suggested that approximately 6 mA current applied to the scalp would be needed to reach 1 mV/mm voltage gradient in the living brain (Fig. 5d; Pearson’s linear correlation; *R* = 0.80; *P* < 0.001; *n* = 16). Across all experiments in which scalp, skull, and epidural stimulations were tested (*n* = 6), we could establish that 58 ± 7% of the current applied through the scalp diffused through the soft tissue surrounding the head. Another 16 ± 8% of the current was attenuated by the resistance of the skull, whereas current spread effectively in the brain, including the meninges, vasculature, ventricles, gray matter, and white matter (Fig. 5e, f; 0.12 (IQR = 0.07–0.19) and 0.62 (IQR = 0.44–0.79) mV/mm/mA for transcutaneous vs. subcutaneous comparison; 0.61 (IQR = 0.49–0.80) and 0.93 (IQR = 0.67–1.23) mV/mm/mA for subcutaneous vs. epidural comparison; paired *t*-test; *P* < 0.001 in both comparisons; *n* = 36 and 60 for the two comparisons, respectively), supporting the view that the brain is an effective volume conductor^3^. Skull thickness was a potential factor in attenuation of the current spread, explaining some of the variability across subjects (Fig. 5g; Supplementary Figure 8; Pearson’s linear correlation; *P* = 0.008; *R*^2^_adjusted_ = 0.046; *n* = 128 electric field strength and skull thickness value pairs).

The experiments on the cadaver brains were performed from 3 to 8 days after death (Supplementary Table 1). Water density measurements of brain specimens revealed that the postmortem age had little effect on the hydration level of the brain (Supplementary Figure 9a). However, previous papers reported that the resistivity is increased in the postmortem skull and soft tissues^37,38^. To mimic the effects of postmortem changes on our cadaver experiments, we simulated the effects of changing the resistance of the soft tissue and examined the role of shunted current by soft tissue (parallel resistance) and the current entering through the skull (serial resistance). Our simulations showed that the lower resistances of the live soft tissues shunt the scalp-applied currents even more than in the cadavers (Supplementary Figure 9b, c).

Overall, our measurements in human cadavers demonstrate that a significant fraction of the scalp-applied current is lost due to the shunting effects of the skin and soft tissue and the serial resistance of the skull. Approximately three quarters of the current was attenuated across the scalp and skull. These findings were further supported by measuring the induced voltage fields first in vivo, followed by identical measurements up to 5 postmortem days in chronically implanted rats (*n* = 3; Supplementary Figure 9d).

### Affecting human brain network activity by ISP

**Fig. 6:**
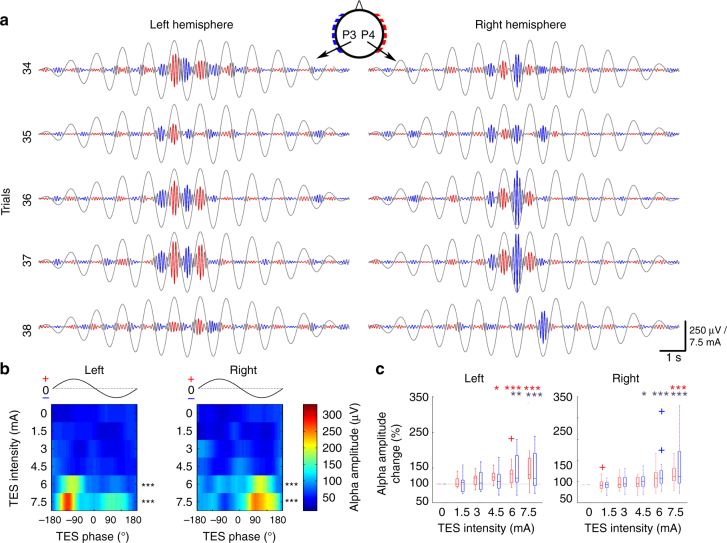
ISP stimulation of the scalp phasically modulates ongoing brain oscillations in human subjects. **a** Five consecutive example trials demonstrating alpha amplitude increase for high-intensity ISP stimulation. Alpha-band filtered EEG signals are color coded based on the instantaneous ISP phase for better visibility; blue and red colors denote stimulus trough (right-to-left currents) and peak (left-to-right currents), respectively. Gray sinusoids denote the ISP stimulus epoch with an increasing–decreasing amplitude. **b** Phase modulation of the alpha amplitude by ISP stimulation for the entire session from the same subject as shown in **a**, showing intensity-dependent alpha amplitude increase (mean across phases are tested in *n* = 45 trials against 0 mA condition, *P* < 0.001 for 6 and 7.5 mA). Note the alternating phase modulation of the left and right hemisphere-derived EEG signals at 6 and 7.5 mA intensities. Color maps show the phase-dependent median alpha amplitudes. **c** Population analysis for the left and right hemispheres, respectively, revealed an intensity-dependent effect. Alpha amplitudes at stimulus peaks and troughs were generally unchanged for stimulus intensities below 4.5 mA. In response to anodal currents in the same hemisphere, phasic modulation was significant at 4.5, 6, and 7.5 mA. In response to cathodal stimulation in the contralateral hemisphere, significant effects were observed only at 7.5 mA (right hemisphere) or 6 and 7.5 mA (left hemisphere)

Our human cadaver and in vivo rat measurements indicated that approximately 6 mA currents applied to the scalp are needed in humans to effectively alter brain networks. To test this prediction, a circular array of 12 stimulation electrodes (six on each side; Fig. 5a) was placed around the head and an ISP protocol was applied in 19 healthy human subjects. Each stimulation site consisted of a 0.9% NaCl solution-soaked sponge square connected to 2 × 3 cm copper mesh. Scalp EEG was monitored by a 2-site montage (P3 and P4 against reference Pz). In each session, 1-min long control recordings were obtained before and after the stimulation session (12 min). To avoid onset and offset effects, ISP stimulation consisted of a train of 1-Hz sinusoids with increasing and decreasing intensity (0, 1.5, 3, 4.5, 6, 7.5, 6, 4.5, 3, 1.5, 0 mA per cycle; Fig. 6a, b; Supplementary Figure 2b) for 12 s, repeated 60 times for each subject. The low frequency (1 Hz) stimulation allowed us to investigate the anodal–cathodal phase modulation of the amplitude of the spontaneous EEG (represented mainly by the dominant alpha band activity) simultaneously in the left and right hemispheres. This approach exploits the observation that TES can coherently affect the membrane potential (*V*_m_) of many similarly oriented neurons (Fig. 2) and the one order of magnitude lower stimulation frequency compared to the oscillation being observed reduces the possibility that stimulation artifacts and their harmonics contaminate the results^4^. The residual ISP artifacts were removed by an offline subtraction of the stimulation-triggered moving average (Supplementary Figure 1a). The artifact-removed signal preserved the major features of the unstimulated control brain activity, as demonstrated by the non-zero peaked cross-correlograms and the weakly correlated instantaneous frequencies in the alpha band between the two EEG channels (Supplementary Figure 1b, c).

TES phase modulation of the amplitude of alpha waves became visible by eye on the filtered signal at high ISP intensities (6 and 7.5 mA; Fig. 6a, b; paired *t*-test with Bonferroni correction; *P* < 0.001 for 6 and 7.5 mA in both hemispheres; *n* = 45 trials). The LFP modulation was present in both hemispheres and alternated in phase, due to the shifting of the anodal–cathodal current direction (compare blue and red epochs in Fig. 6a, b). For group statistics, the mean alpha amplitudes near the stimulus peak (−135° to −45°) and near the stimulus trough (45° to 135°) were measured separately at P3 and P4 at each current intensity. Significant modulation of the LFP amplitude by the TES phase was observed at current intensities of 4.5, 6, and 7.5 mA at each hemisphere when the preferred current direction was applied (Fig. 6c, paired *t*-test with Bonferroni correction; left hemisphere at TES trough: *P* = 0.006, <0.001 for 6 and 7.5 mA; left hemisphere at TES peak: *P* = 0.01, <0.001, <0.001 for 4.5, 6, 7.5 mA; right hemisphere at TES peak: *P* < 0.001 at 7.5 mA; right hemisphere TES trough: 0.01, <0.001, <0.001 for 4.5, 6, 7.5 mA; *n* = 1025 trials for all conditions from 18 subjects. All intensities were tested against the 0 mA condition).

Stimulation intensity of 4.5 mA or higher induced various self-reported subjective effects, including tingling and slight burning feeling of the skin in each subject. Subjective feeling of head-movement (dizziness) and horizontally oscillating light in the visual field at the frequency of the stimulation was often reported even though the eyes were closed and testing was performed in semi-darkness. Feeling of a moving noise source in the horizontal plane at 1 Hz was also reported. Nearly all subjects also reported a ‘metallic taste’ in the mouth. All subjective effects were stronger at the beginning of the stimulation session and attenuated (though they did not disappear) by the end of the session. No post-stimulation aftereffects were reported after any session. One subject requested an early termination of the stimulation due to dizziness and their data were excluded from the study.

**Fig. 7:**
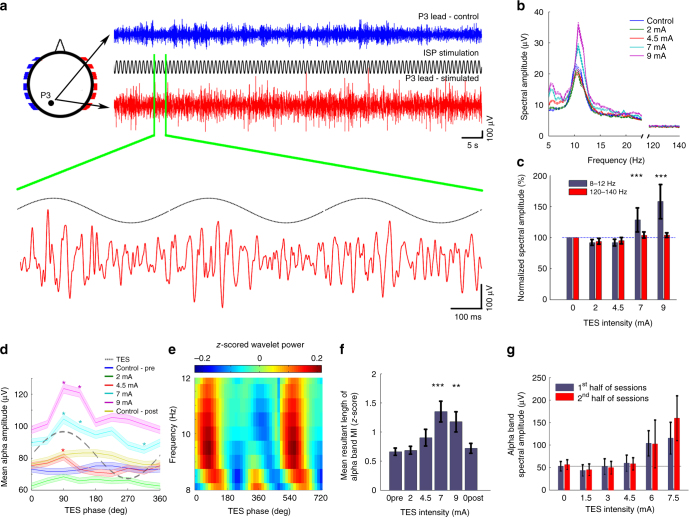
High intensity ISP stimulation of the scalp phasically modulates ongoing alpha waves in human subjects. **a** Blue, control EEG trace; red, EEG trace during 7 mA stimulation (eyes closed in each condition). A 3-s magnified segment of EEG trace at P3 lead is also shown. Note the absence of signal saturation. The 1 Hz modulation of the baseline was removed. **b** Single session example of power spectra of EEG traces during increasing ISP intensities at 1 Hz. Power spectra were calculated in 10-s long windows, then averaged. **c** Quantification of ISP stimulation-induced increase in alpha band power in a single session. The control frequency band (120–140 Hz) showed no significant change (*n* = 405–408 stimulus cycles; *P*_alphaband_ = 0.37, 0.42, <0.001, <0.001; *P*_control_ = 1.38, 1.38, 0.31, 0.62 for 2, 4.5, 7, 9 mA; all vs. 0 mA). **d** Single session example of alpha wave amplitudes as a function of the phase of 1 Hz ISP sinusoid stimulation. Asterisks denote phase bins significantly different from the mean (one-way ANOVA; *P* < 0.05 at *F*(7, 3140) = 3.033 for 4.5 mA, *F*(7, 3136) = 6.96 for 7 mA, and *F*(7, 3160) = 14.37 for 9 mA; asterisks show significant (*P* < 0.05) phase-intensity combinations of the post-hoc *t*-tests against the phase bins of the control condition. **e** Single session example wavelet map (9 mA, 1 Hz ISP) shows ISP phase modulation of the alpha band power. **f** Alpha band power modulation of wavelet decomposed EEG by 1 Hz ISP stimulation phase (*n* = 16, 10, 8, 18, 10, and 12 sessions; *P* = 0.98, 0.041, <0.001, 0.019, and 0.17 for ‘0pre’, 2, 4.5, 7, 9, and ‘0post’ intensities in 3 subjects; two-sample Kolmogorov–Smirnov test). **g** ISP stimulation-induced increase of alpha power was stable throughout the recording epochs, as shown by the similar values during the first and second halves of the stimulation sessions (*n* = 23 trials from a single subject, *P* = 0.96, 0.79, 0.44, 0.44, 0.74, and 0.11 for 0, 1.5, 3, 4.5, 6, and 7.5 mA intensities, respectively)

In three of the subjects, we also used step currents of 1 Hz ISP stimulation, instead of the intensity increasing–decreasing ramp. The results of this experiment support those obtained by ramp stimulation (Fig. 7a–f). Current intensity exceeding 4.5 mA increased alpha band spectral amplitude (Fig. 7b–c; paired *t*-test with Bonferroni correction; *P* < 0.001 for 7 and 9 mA vs. 0 mA; *n* = 405–408 power values for each stimulus intensity), and brought about stimulus phase-dependent amplitude modulation of the alpha waves (Fig. 7d–f; two-sample Kolmogorov–Smirnov test; *P* < 0.001 and 0.019 for 7 and 9 mA vs. 0 mA; *n *= 16, 10, 8, 18, 10, and 12 modulation vector length). This experiment also showed that the subjective decrease of the perceived sensory effects over the course of the experiments cannot be attributed to changes in alpha power since alpha power did not change between the first and second halves of the experiment (Fig. 7g; paired *t*-test; *P* = 0.96, 0.79, 0.44, 0.44, 0.74, 0.11 for 0, 1.5, 3, 4.5, 6, 7.5 mA intensities; *n* = 23 spectral amplitude pairs).

**Fig. 8:**
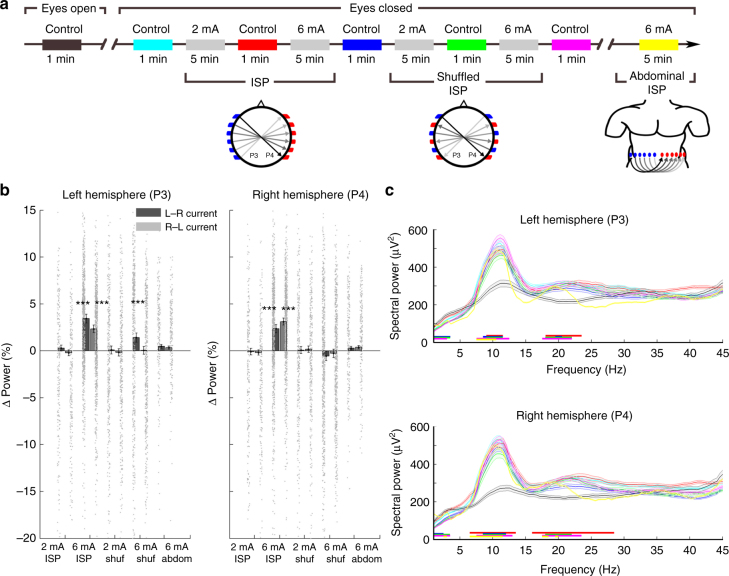
Comparison of ISP and shuffled ISP stimulation on the EEG of human subjects. **a** Testing sequence of the experimental protocol. ISP stimulation used the same arrangement as in Fig. 5. During shuffled ISP, adjacent stimulation electrodes were stimulated with opposite polarity. While shuffled ISP increases local current flow in the scalp, the alternating directions of the induced electric fields at the focus area are expected to cancel, ideally resulting in a zero current in the brain. **b** Group results shown separately for the left and right hemispheres. Six mA current ISP stimulation increased alpha power in both hemispheres. Shuffled ISP exerted only a unilateral and weaker effect. Intensities at 2 mA were ineffective. Abdominal stimulation (6 mA ISP protocol) did not exert a significant effect on alpha power (*n* = 809 epochs; power difference values for each conditions from 6 subjects, one-sample *t*-test with Bonferroni correction). **c** Spectral power comparison between eyes open control and eyes closed control periods. Horizontal lines indicate significant changes from the eyes open condition (*P* < 0.05; *n* = 125 epochs for the eyes open, 144, 117, 126, 127, 148 epochs for the consecutive eyes closed, and 211 epochs for the abdominal ISP conditions; Mann–Whitney *U*-test with Bonferroni correction). Color coding of the conditions is the same as in **a**

In a separate group of subjects (*n* = 6, Supplementary Table 4), we examined whether the EEG changes could be accounted by non-specific sensory stimulation or potential arousing effects of TES. We used current steps (tACS) of 1 Hz ISP stimulation (2 or 6 mA for 5 min with 1-min rest periods; Fig. 8a). To test for the effects of peripheral sensory stimulation, we interleaved the ISP stimulation epochs with a similar protocol, where the adjacent electrode pairs were stimulated with opposite polarity (‘shuffled ISP’ protocol—first pair received an L–R direction current pulse, second one received an R–L, third one an L–R, and so on; Fig. 8a). Because of the alternating direction of the induced electric fields with the shuffled ISP, the summed effect in neurons was expected to be close to zero. The results with the regular ISP protocol confirmed the hemisphere-specific, stimulation phase-induced modulation of alpha waves (Fig. 8b; Supplementary Figure 10; one-sample *t*-test with Bonferroni correction; *P* < 0.005 for L–R and R–L direction 6 mA ISP in both hemispheres; *n* = 809 power difference values). In contrast, the shuffled ISP induced only minor physiological effects on EEG activity (Fig. 8b; one-sample *t*-test with Bonferroni correction; *P* < 0.005 for L–R 6 mA shuffled ISP in left hemisphere; *n* = 809 power difference values). Stimulus intensity at 2 mA failed to induce any detectable changes (ISP or shuffled ISP; one-sample *t*-test with Bonferroni correction; *P* = 0.36, 1.06, 2.22, and 1.45; *n* = 809 power difference values). As an additional control for potential arousal effects, the same subjects were also tested with the same ISP protocol but with the stimulation electrodes placed on the abdominal wall (Fig. 8a). No hemisphere-specific, stimulation phase-induced modulation of alpha waves was observed in the peripheral control experiment (Fig. 8b; 6 mA abdominal; one-sample *t*-test with Bonferroni correction; *P* = 0.41, 0.39, 1.68, and 1.18; *n* = 809 power difference values).

The self-reported subjective effects in this last experiment, using the ISP protocol with step increase of tACS, were similar to those observed with the ramp stimulation (Supplementary Table 4). In contrast, under the shuffled or the abdominal ISP protocol no subject reported phosphenes, vestibular effects, or metallic taste. On the other hand, each subject judged that the feeling of skin ‘burning’ was stronger under the shuffled ISP protocol. No post-stimulation aftereffects were reported by any subject.

Finally, we asked whether the ISP or shuffled ISP stimulation exerted a long-lasting blocking effect on occipital alpha oscillations. Alpha dominance of the EEG was present after each stimulation period (eyes closed) and each period was significantly different from the control pre-stimulation eyes open condition (Fig. 8c; Mann–Whitney *U*-test with Bonferroni correction; *P* < 0.05; *n* = 125–148 spectral power values for each of the 500 discrete frequencies). Some periods also showed significant increase in beta power (16–28 Hz) and decreased power in the delta (<4 Hz) band compared to the eyes open condition. None of the post-stimulation periods were different from the pre-stimulation eyes closed control condition. Overall, these experiments showed that ISP stimulation at sufficient stimulus intensity (4.5–6 mA) can bring about stimulus phase-dependent amplitude modulation of the EEG, an effect which cannot be attributed to peripheral sensory or arousal effects.

## Discussion

Using in vivo experiments in rodents and high-density intracerebral recordings in human cadaver brains, we found that scalp-applied currents were halved by the skin and subcutaneous soft tissue and further attenuated by the skull by 16%. To reduce the scalp stimulation side effects and enable recording of brain activity during stimulation, we designed an ISP stimulation method that allowed injection of higher current intensities into the brain compared to conventional tACS and transcranial direct current stimulation (tDCS) approaches, while keeping the cumulative charge density on the scalp surface and sensation relatively low. The spatial specificity of the ISP method was demonstrated by hemisphere-specific activation of single neurons in rats. Finally, we show that sufficiently strong induced fields can directly and instantaneously affect brain networks in healthy subjects.

Neuronal excitability is largely determined by ionic conductances brought about by neurotransmitter-induced postsynaptic potentials. However, neurons can also sense electric fields directly^1–3,28,39^. In principle, the effectiveness of the externally applied field depends on a variety of factors including neuronal density and geometry, alignment of dendrites and axons relative to the induced field, type and distribution of ion channels in the neurons, degree of myelination, and density of glia, which can affect the electric shunting effect of the extracellular space. These factors can change between brain regions and might vary across species and their potential significance must be addressed experimentally. The additive nature of the two polarizing mechanisms (synapse-mediated and ephaptic) can be exploited to influence subthreshold membrane potentials and spiking^1^. When a neuron is about to emit a spike, even a weak electric field can bias spike threshold. In vitro experiments have shown that coupling an oscillatory field to intracellularly generated oscillation can be effective with as small as 0.2 mV/mm gradient^40^ and 1 mV/mm can induce measurable subthreshold *V*_m_ effects and affected timing of action potentials^39^. Whether such weak stimuli, applied acutely or chronically, can have beneficial or deleterious effects on brain function can only be determined by targeted recordings and additional behavioral measures. To place such weak forced fields into perspective, voltage gradients of >4 mV/mm are brought about by hippocampal theta oscillations across the CA1 pyramidal layer and >15 mV/mm fields are induced by sharp waves^1^. However, to exert a reproducible and instantaneous impact on local networks by forced fields, the discharge of at least a fraction of neurons with common targets should be temporally coordinated. Our in vivo intracellular recordings have revealed that electric fields >1 mV/mm were needed to exert measurable effects on spikes and subthreshold *V*_m_, but several times larger currents were required to measurably affect the associated network rhythms^1,4,6,8,12,41^. This difference may be explained by the competition between the applied fields and the strong influence of endogenous network patterns.

Given the complex paths of current spread in the brain^42^ and the importance of neuronal geometry in sensing fields^43^, the absence of an effect in any experiment cannot be taken as evidence for absence of effects on a few neurons. Yet, it is also important to emphasize that the requirements of affecting spike threshold of some neurons occasionally in wide areas of the brain vs. consistently biasing activity of neuronal circuits are different. We focused our efforts on establishing a current intensity level required to reliably affect local networks in the intact brain. The findings demonstrate that to consistently affect spikes and local networks, sufficient magnitude of charges has to be applied to the scalp to achieve >1 mV/mm voltage gradient^44^. In our rodent experiments, more neurons were excited than suppressed by alternating current simulation, and the depolarizing fields were more effective than hyperpolarizing fields. These findings illustrate that externally applied alternating fields bring about a dominant excitatory gain. We emphasize though that our results cannot rule out the potential impact of long-lasting (minutes to hours) weak electric fields on behavior by yet unidentified mechanisms^45^.

Although computational methods have become increasingly sophisticated over the years^17,28,35^, experimental data are needed to justify the modeling assumptions. Subdural measurements in implanted patients^46^ can be useful but limited for estimating the currents necessary to bias spiking because they estimate fields tangentially to the cortical surface, whereas the largest voltage gradients are oriented orthogonal to the cortical surface^44^. Using scalp, cranial, and epidural stimulation electrodes and multiple recording electrodes, we quantified the 3-D spread of electric fields in both rodents and human cadavers. Our findings confirm the largely ohmic nature of current spread in the brain^34^, skull, and the surrounding soft tissue^35^. The scalp, subcutaneous tissue, and muscles function as an effective shunt, resulting in at least 50% reduction of applied current intensity. The serial resistance of the skull further reduces the current flow by another 10–25%, depending on the thickness of the skull^47^. Given the importance of these attenuating factors, the amount of soft tissue, hair, and skull thickness should be taken into account in estimating the effective current reaching the brain^35^, and variation of these factors may explain the large individual variability of the TES-induced effects^14,46,48^.

In TES practice, currents larger than 2 mA are avoided because higher intensities are associated with skin sensation, phosphenes, and other side effects^7,9,49^. Our intracellular and extracellular measurements in rats, along with previously published data^4,6^ and models^17,41^, suggest that 1 mV/mm is needed to affect neuronal firing consistently and even larger strengths may be needed to phase-entrain brain rhythms to arbitrary stimulus frequencies. In our cadaver experiments, we found that scalp-applied currents should exceed 4–6 mA to achieve 1 mV/mm voltage gradient in brain tissue but direct comparison with in vivo brain measurements are not available. Electric conductivity of the postmortem tissue may change after death. However, our in vivo vs. postmortem comparison in rats demonstrated that in the intact head, actually larger currents are needed to achieve voltage gradients comparable to those of the cadaver experiments, possibly due to the stronger shunting effect of the better hydrated in vivo tissue^38^.

To reduce scalp sensation or other side effects^31,41^ and to increase the direct effects of TES on brain activity^7,9–14^, novel approaches are needed^18^. Our ISP method exploits the time-integrating property of the neuronal membrane (i.e., the membrane time constant of neurons is ~10 ms), by applying brief and rapidly rotating fields. Using the ISP protocol, the strongest transmembrane charge builds up where successively induced electric fields (‘beams’) intersect, although in the human brain the anisotropy brought about the cortical gyri and ventricles should be taken into consideration. The more ‘beams’ are used, the smaller the adverse effects on other areas. Using just three rotating dipoles in rats, we demonstrated a proof of principle for the focusing effect of ISP by confining the ISP effect to largely one hemisphere. In addition, when adjacent electrodes were stimulated with opposite polarity in human experiments (shuffled ISP), no reliable brain responses were detected, even though skin sensation side effects increased. The latter effect may be explained by the stronger current density induced by the opposite polarity of neighboring sites.

An experimental advantage of the ISP technique is that high frequency pulses do not saturate recording amplifiers. This feature allowed us to measure the physiological effects of scalp stimulation in human subjects. Instead of focusing on brain rhythm-entrainment effects^15,50^, in which residual artifacts are notoriously difficult to eliminate^15,23^, we examined how the amplitude of the spontaneous LFP was biased by the slowly changing forced fields^4^. This method is similar to using tDCS at multiple current levels, where the additive/subtractive effect of the applied field can be probed on the amplitude of native network patterns^4^. We have verified the validity of this approach previously in rodents, using both LFP and unit firing^4^. In support of the estimated voltage gradients from the cadaver experiments and the ‘minimum’ fields (~1 mV/mm) in rodents to affect network activity, we found that >4.5 mA currents were required to reliably bias the amplitude of occipital alpha waves. While we designed our experiments to maximize the stimulation effects on the parietal–occipital region where alpha waves are of largest amplitude, we cannot exclude the possibility that an improved configuration of stimulation sites could reduce the minimum effective current somewhat further in future studies.

In our human experiments, we used six pairs of scalp stimulation electrodes, reducing the required local momentary current by six-fold. Still, the effective intensities needed to directly bias intracerebral neuronal activity induced adverse skin effects and vestibular reactions. An obvious next step in advancing the ISP technique is to increase the number of intersecting dipoles generated by pairs of stimulating electrodes. For example, using a montage of 32 electrodes with highly conductive coupling to the skin, a large number of dipoles can be formed to create a circumscribed 3-D intersectional focus or target two or more brain structures while reducing the locally applied currents, potentially below the skin sensation threshold. Combining ‘ground truth’ measurements from the human cadaver brain with computational models of the head can lead to a rationale design of focused electric activation of brain structures without adverse and perceivable peripheral effects.

In conclusion, we demonstrate that affecting neuronal circuits directly and instantaneously in the human brain requires higher intensity currents than used in conventional TES experiments. Implicitly, our results also suggest that behavioral and cognitive effects reported in previous tACS studies have likely been achieved by indirect mechanisms on brain activity, which needs to be explored in detail. To achieve sufficient magnitudes of intracranial fields without direct peripheral side effects, novel methods will be required.

## Methods

### Ethical permissions

The experiments were approved by the Institutional Animal Care and Use Committee of New York University Medical Center IACUC (protocol number: 160926–01), the Ethical Committee for Animal Research (ethical permission numbers: XIV/471/2012 and XIV/218/2016), and the Ethical Committee for Human Research (ethical permission numbers: 98/2013 and 164/2014, for the measurements on cadavers and healthy subjects, respectively) at the Albert Szent-Györgyi Medical and Pharmaceutical Center of the University of Szeged in accordance with European Union guidelines (2003/65/CE).

### Experiments on rats

A total of 16 female, 3 male Long-Evans rats (350–450 g, 10–16 weeks old) and 8 male Wistar rats (250–450 g, 8–12 weeks old) were implanted with custom-made recording and stimulating electrodes under urethane anesthesia (1.3–1.5 g/kg, intraperitoneal injection) for the extracellular and the whole-cell patch clamp recording experiments, respectively. Sample size for each experiment was estimated on the basis of anticipated inter-animal neurophysiological variability and the expected high success rate of the experiments in terms of the number of recorded neurons and data quality based on our previous studies. No rats were excluded from the analysis. Each animal served as its own control, no randomization or blinding was employed. Rats were kept on a regular 12 h–12 h light–dark cycle and housed in pairs before implantation. No prior experimentation had been performed on these rats. After anesthesia induction, atropine (0.05 mg/kg, s.c.) was administered to reduce salivation, and the rectal temperature was kept constant at 36–37 °C with a DC temperature controller (TMP-5b; Supertech, Pécs, Hungary). Stages of anesthesia were maintained by confirming the lack of vibrissae movements and nociceptive reflex. Skin of the head was shaved and the remaining fur was completely removed by using depilatory cream.

### Recording intracerebral electric fields in anesthetized rats

To record the stimulus-induced intracerebral electric fields, the skin was retracted after a mediosagittal incision, and the bone surface was cleaned and dried as described before^51^. Two strips of three custom-molded silicon pockets (2-by-2-by-1 mm, 4 mm^2^ surface area, 1.2 mm spacing) were glued onto the temporal bone bilaterally, and a single pocket above the prefrontal bone (2 mm anterior from bregma in the midline) using cyano-acrylic glue. Pockets were filled with conductive paste (Super Visc, Brain Products, Germany) and then sealed with silicon. An Ag/AgCl reference electrode was placed in the subcutaneous space behind the neck. Thirty holes (0.5 mm diameter) were drilled in the skull and a custom-made 6 × 5 recording electrode matrix was inserted into the brain 3 mm deep below the brain surface (Fig. 1a). The spacing between the individual electrodes was 2, 1.7, 2.2, 1.7, and 2 mm in the *x* axis and 2 mm in the *y* axis. Each recording electrode was made by a polyurethane-insulated, copper–nickel wire (50 µm diameter) inserted into a supporting polyimide tube (70 µm interior diameter, 86 µm outside diameter).

Subcutaneous tACS was performed in voltage controlled mode, generated by an STG4008–16mA (Multi Channel Systems, Reutlingen). To monitor the applied current, a 100 Ω resistor was placed in series with the stimulating electrodes and the voltage drop were measured across the resistor by an isolation amplifier circuit to preserve the ground-independent stimulation configuration. The prefrontal electrode was used as an anode and the lateral ones served as cathodes. Varying frequencies (10, 20, 50, 100, 200, 500, 1000, and 2000 Hz) at 3 V were delivered through the stimulating electrodes paired in various configurations. Recorded signals (n = 30 channels) were amplified (10× gain) and stored after digitization at 20 kHz sampling rate per channel (RHD2000 Evaluation System, Intan Technologies, Los Angeles).

### Measuring the spatial selectivity of focused ISP in vivo

Two custom-designed (AutoCad, San Rafael, CA, USA) stimulation strips were 3-D printed (Form 1+, Formlabs, Somerville, MA, USA) and glued bilaterally on the surfaces of the temporal bones of the rats by cyano-acrylic glue (Loctite, Henkel). Each of the two symmetric strips (width 13 mm, height 3.3 mm, and wall thickness 0.7 mm) consisted of five individual pockets which were separated from each other by 3.7, 2.2, 2.2, and 3.7 mm (Fig. 2b), and their medial surfaces were resembling the temporal bone curvature of a magnetic resonance imaging (MRI) data-based 3D model of a rat skull. The middle pockets were positioned at 5.16 mm posterior from bregma. The pockets were filled with conductive paste through filling holes left open at the top (Super Visc, Brain Products, Germany) and then sealed with silicon. Craniotomies were drilled (2.2 mm diameter) and two silicon probes (Buzsaki32-H32; NeuroNexus, Ann Arbor, MI, USA) were implanted at 5.16 mm posterior from bregma and 4 mm lateral of the midline, in the CA1 region of the hippocampus. The hole around the probes was filled with non-conductive silicon (Dow Corning®, Midland, MI, USA). Proper locations of the electrodes were confirmed by the characteristic electrophysiological landmarks of the broadband signal at the pyramidal layer of CA1. ISP stimulation was performed in a voltage-controlled mode using phototransistor-based custom-made electronics described below.

### Comparing the effect of TES and ISP stimulation

To compare the effects of ISP and DC stimulation in rats, the same surgery procedure was applied but the stimulation was performed in current-controlled mode (stimulus intensity 200 µA) using the high-speed analog switch-based circuits described below. The recorded signals (*n* = 64 channels) were amplified (400× gain) and stored after digitization at 20 kHz sampling rate per channel (KJE-1001, Amplipex, Szeged, Hungary). We repeated the same measurements on one awake, freely moving animal^4^.

### Comparing transcutaneous and subcutaneous stimulation

For transcutaneous electrical stimulations, a pair of silicon single-pocket electrodes filled with conductive EEG gel was glued on both sides of the head of the rats, as described above (Fig. 1c). Small incision on the scalp was then made for craniotomy after subcutaneous lidocaine injections. Small craniotomy was drilled (1.2 mm diameter) and a silicon probe (Buzsaki32-H32; NeuroNexus, Ann Arbor, MI, USA) was inserted in the axis of the stimulating electrodes at 3 mm posterior from bregma and 2 mm lateral of the midline, into the CA1 region of the hippocampus. A 50-µm insulated wire electrode (California Fine Wire, Grover Beach, CA, USA) was attached 1.2 mm away from the fourth shank serving as a reference electrode. The hole around the probe was filled in with silicon (Dow Corning®, Midland, MI, USA).

After the transcutaneous stimulation, the silicon probe was removed and the skin was retracted and another set of silicon pocket electrodes were attached onto the temporal bone, as described above. The silicon probe was inserted again at almost the same location (2.8 mm posterior from bregma and 2 mm lateral of the midline).

Varying frequencies (10, 100, and 1000 Hz) at varying amplitudes (10, 20, 50, 100, and 200 µA) were used for both settings in current-controlled mode (STG4002; Multi Channel Systems, Reutlingen, Germany).

The recorded signals (*n* = 32 channels) were amplified (400× gain) and stored after digitization at 20 kHz sampling rate per channel (KJE-1001, Amplipex, Szeged, Hungary).

### Measuring the effect of postmortem age

Rats were implanted with a pair of silicon pocket electrodes as described above. Twelve holes (0.5 mm diameter) were drilled in the skull and a custom-made 6 × 2 recording electrode matrix was inserted into the brain. The spacing between the individual electrodes was 2, 1.7, 2.2, 1.7, and 2 mm in the *x* axis and 2 mm in the *y* axis. The electrode matrix was inserted at 3 mm depth in the brain and the craniotomies were filled with silicon (Dow Corning®, Midland, MI, USA). Once the silicon dried, the whole skull was covered by dental cement (Duracryl™ Plus, Spofa Dental, Jičín, Czechia) and the skin was closed by sutures to restore its conductive integrity. Subcutaneous tACS was performed in voltage-controlled mode using various stimulation parameters as described above (STG4002; Multi Channel Systems, Reutlingen, Germany).

After the in vivo measurement, the rats were euthanized by sodium pentobarbital (150 mg/kg, intraperitoneal injection). The corpses were kept at 4 °C after death in plastic bags to prevent desiccation. Subcutaneous tACS and recordings were repeated on postmortem day 1, 2, 3, 4, and 5. In one case, the implantation was done 5 days after the euthanasia, which resulted in qualitatively and quantitatively similar results (data not shown).

### In vivo whole-cell patch clamp recordings

A pair of silicon pocket electrodes filled with conductive EEG gel were glued bilaterally on the skin or on the temporal bone of rats for transcutaneous and subcutaneous stimulation, respectively, similarly to extracellular recording experiments (Fig. 1c). A small craniotomy (~2 mm diameter) was made 5.0 mm posterior from the bregma, 4.0 mm lateral of the midline. Patch-pipettes were made from borosilicate glass capillaries (GC150TF-10; Harvard Apparatus, Holliston, MA, USA) and their tip resistance were 5–7 MΩ when filled with an intracellular solution: (in mM) 135 K-gluconate, 10 HEPES, 10 Na_2_-phosphocreatine, 4 KCl, 4 ATP-Mg, and 0.3 GTP-Na_3_ (pH = 7.25, 290 mOsm). Liquid junction potential calculated as +18.6 mV was offline-compensated. The patch-pipettes attached to a fine stepper motor manipulator were lowered perpendicularly and blind in vivo whole-cell recordings from cortical neurons (0.5–1.3 mm from the pia) were obtained as previously described^52^. Recordings were performed using an EPC10 USB amplifier (HEKA Elektronik, Lambrecht/Pfalz, Germany) with PATCHMASTER software (ver. 2.901 HEKA Elektronik). Signals were filtered at 3 kHz and digitized at 20 kHz. The pipette capacitance, membrane capacitance, and series resistance were compensated. If series resistance varied more than 20% or increased above 50 MΩ, the data were discarded. Isolated direct constant current stimuli (~ ±800 µA) were delivered via a multifunction stimulator (STG4002; Multi Channel Systems, Reutlingen, Germany). After the whole-cell transmembrane potential recordings, the recorded neurons were detached from the pipette with retraction and positive pressures. After retraction, the artifacts of the same set of electrical stimuli applied during the whole-cell recordings were recorded extracellularly. The recorded artifacts were subtracted from intracellularly recorded potentials to recover the true transmembrane potentials^25^. Finally, a 4-shank 32-channel silicon probe (Buzsaki32-H32; NeuroNexus, Ann Arbor, MI, USA) was inserted in the vicinity of the recorded neuron to record extracellular electrical gradients in response to the same stimulation as during intracellular recordings. The extracellular recordings were performed at 20 kHz sampling rate using a KJE-1001 amplifier (Amplipex, Szeged, Hungary).

### Measurements on human cadavers

Recordings were performed at the Department of Pathology, Faculty of Medicine, University of Szeged. Medical history of cadavers was consulted in advance and only those with no known brain disorder were selected for measurements. The corpses were kept at 4 °C after death in plastic bags until autopsy to prevent desiccation. The autopsy theater temperature was 22 °C. The routine medical autopsy procedure was done on the same day as experimental measurements. The sample size required (number of cadavers and recording sessions) was extrapolated from the results of animal studies. There was no blinding or randomization employed. No cadaver was excluded from the analysis.

### Recording tACS-induced intracerebral electric fields

The scalp was cut along the coronal plane connecting the mastoids. The anterior and posterior halves of the scalp were retracted forward and backward, respectively, until the supraorbital ridge and the occipital protuberance was revealed. The temporal muscles and soft tissue were also removed. After the skull was cleaned, the head was fixed in a custom-made acrylic glass frame (Supplementary Figure 3). The top of the skull was pushed against the acrylic frame as close as possible. Four stainless-steel screw bars (6 mm diameter, 10 cm length) held the head steady on each side. One end of the screw bar was attached to the tower by a hexagonal nut, the other end held a rubber ring (3 cm diameter) against the skull. Once the head was positioned, the positions of the 36 penetration holes were marked by an ink-filled needle through the pre-made hole-matrix of the plexiglass back panel. The frame was removed and holes were drilled (1.2 mm diameter) and rinsed by physiologic saline. The frame was placed back to its original position, and the head was repositioned by the screw bars. Four or seven pairs of stimulation electrodes (Ag/AgCl EEG electrodes, 10 mm diameter, Ambu, Copenhagen, Denmark) were placed between the rubber rings and the skull surface with conductive paste (Ten20, D.O. Weaver, Aurora, CO, USA). Custom-made multiple-site electrodes were prepared as follows: three to seven holes were drilled on the outer surface of a translucent polyimide tube (724 μm internal diameter, 775 μm outside diameter). Three to seven 127-μm diameter, polyurethane-insulated, copper–nickel wires were threaded into the polyimide tube through these holes space 1 cm from each other. The wires were secured by a drop of cyano-acrylic glue at the side-holes of the polyimide tube and the other end of it was soldered to a connector socket. The tubes were backfilled with epoxy glue to increase stiffness. Once the epoxy dried, the wires were cut back at the surface of the polyimide tube, and the tip of the tube was sharpened. Impedances of the contact sites varied between 50 and 300 kΩ at 1 kHz. Electrodes were inserted into the brain through the previously drilled skull holes and the matching plexiglass matrix while rotating continuously, to preserve parallel alignment.

A needle was inserted through the skull above the prefrontal cortex and served as reference electrode. Physiologic saline solution (2–5 ml) was injected through the same hole to refill the cerebrospinal fluid lost during the drilling procedure. Recording electrodes made a watertight seal in the skull holes, thus further leakage was not significant. The chest wall was used as grounding. Subcutaneous (electrodes placed on the skull surface) alternating and direct current stimulation was performed using stimulation signals generated by either an STG 4008–16 mA (Multi Channel Systems, Reutlingen) or an NI 6343 board (National Instruments) with precision isolation amplifier circuits. The floating (cadaver) side of the isolation amplifiers were powered using two 9-V batteries for each stimulator pair. To monitor the applied current, a 100-Ω resistor was placed in series with the stimulating electrodes and the voltage drop across the resistor was measured by an isolation amplifier circuit. The stimulating electrodes of the two sides were paired using different parallel or diagonal arrangements. Sinusoid stimuli with varying intensities (1, 2, 3, 4, 5, and 6 V) at 10 Hz and varying frequencies (5, 20, 50, 100, 200, 500, 1000, and 2000 Hz) at 5 V were used for at least 200 cycles, each. To mimic the effect of increasing electrode sizes, multiple stimulating electrodes were coupled together. In some cases, an additional stimulation electrode was placed in the midline of the forehead, and used against a selected lateral electrode to achieve a fronto-lateral stimulation configuration. The recorded signals (*n* = 198 channels) were amplified (10× gain) and stored after digitization at 1.6 kHz sampling rate per channel by a custom-designed circuit (for DC coupled recordings) or at 20 kHz sampling rate per channel by another custom-designed recoding system based on the RHD2000 Evaluation System (for AC coupled recordings, 0.1–6 kHz bandwidth, Intan Technologies, Los Angeles, CA, USA).

### Recording tDCS-induced intracerebral electric fields

Instead of the above-mentioned electrode matrix, six custom-made single contact Ag/AgCl electrodes were introduced in the fourth coronal plane in 2 cadavers. A silver wire (400 µm diameter) was inserted into a translucent polyimide tube. The contact sites were cleaned by scratching with a razor blade and then immersed into NaOCl (42 g/l) for 16 h. The same recording system was used to acquire signals (10× gain, 1.6 kHz sampling rate per channel). All stimulating electrode pairs were active simultaneously and anodal or cathodal stimulation was applied for 50–50 s (5 V intensity).

### Measuring the shunting effect of the skin and skull

Instead of retracting the skin, four or six 5-mm long incisions (15 mm apart from each other) were made on both sides of the sagittal suture in the coronal plane, connecting one mastoid with the other. To prevent soft tissue damage, the drilling head was used through a metal tube (1.3 mm diameter) and four holes were made. Then stimulation electrodes (*n* = 4, Ag/AgCl, Ambu, Copenhagen, Denmark) were attached to the skin by conductive paste (Ten20, D.O. Weaver, Aurora, CO, USA). Four or six custom-made 7 contact site recording electrodes were inserted into the brain, transcutaneous alternating current stimulation was performed, as described above. The recorded signals (*n* = 28 or 42 channels) were amplified (10× gain) and stored after digitization at 15 kHz sampling rate per channel (RHD2000 Evaluation System, Intan Technologies, Los Angeles, CA, USA). After the skin measurements, the skin incisions were carefully connected and the scalp was removed while the recording electrodes were kept in place. The stimulating electrodes were attached to the skull surface and the same stimulation protocol was applied. In separate experiments, to compare the effect of subcutaneous stimulation to intracranial stimulations, in some cases additional stimulating electrodes were placed intracranially, in between the subcutaneous electrodes as follows: the additional skull holes were drilled with incrementally increasing (2, 4 and 8 mm) drill-bit sizes, and externally threaded, hollow plastic dowels (15 mm long, 8 mm diameter, Hettich Furntech, Germany) were introduced in the holes to form an electrical isolation toward the skull. Sponge electrodes with the encapsulated Ag/AgCl plates, soaked in physiologic saline, were glued to the tip of screws, and introduced into the plastic dowels to touch the brain surface.

### Registering the anthropometric data of the cadavers

At the end of the measurements, the cranium was opened with an oscillating saw in the line of the stimulating electrodes. After removing the skull cap, the brain was also removed. Anthropometric data of the skull was measured (circumference, sagittal, horizontal, vertical distance, and skull thickness below the stimulating electrodes). After the brain was examined by the pathologist, a 5-g piece of the occipital lobe was removed to measure the water content of the brain tissue by desiccation. As reference, hydration value of living tissue was taken from reference^53^.

### Measurements on human subjects

Human transcutaneous stimulation and recording experiments were performed on healthy subjects (all males, age = 21–66 years). Subjects with short hair were preferably selected, thus including only males was incidental. All subjects gave their informed consent to the experiments. Each subject served as his/her own control; no randomization or blinding was used.

Before performing the ISP stimulation protocol, each subject was briefly exposed to a few seconds of 1 Hz constant current stimulation with increasing intensities (1, 2, 4, and 8 mA) to familiarize them with the expectable subjective experience during the ISP protocol, and to test if any adverse effects are present. The intensity was increased to the next level only if the previous intensity was reported as being well tolerable. In addition to the well documented tingling, burning feeling of the skin and perception of phosphenes^11^, stimulus intensities above 4.5 mA stimulation induced feeling of horizontal head-movements and horizontal oscillation of the visual and auditory fields at the frequency of the stimulation. All subjective effects were stronger at the beginning of the stimulation and attenuated during the course of stimulation. None of these are considered Serious Adverse Effect or Event by the US Food and Drug Administration^54^. No aftereffects were reported after any session. Phosphenes were likely induced by current spread through the orbits, whereas vestibular and auditory effects were likely due to the spread of currents through the ear canals.

### Considerations of TES effects on human subjects

There are no accepted guidelines about the current limit of either tDCS or tACS^24,49,54^. The main reason for this is the lack of reliable information about the induced fields in the human brain, and this is what we supply in our cadaver studies. Most TES studies use <2 mA, mainly because this is the threshold where peripheral sensation and phosphenes are typically detected. Other related measures include (1) current density (in A/m^2^) at the electrode calculated by taking the applied current to a given electrode and dividing by electrode area and (2) stimulation charge (in Coulombs, C) determined by multiplying current by duration. Since the adverse and risk effects of stimulation are related to current density and duration of stimulation (i.e., the total charge or ‘dose’), 1 mA for 10 min, 2 mA for 5 min, and 10 mA for 1 min are considered equivalent from the perspective of charge^54^, yet these three categories may not be equivalent for subjective side effects or instantaneous direct brain effects. Direct stimulation of the brain via subdural electrodes using 1 ms pulses of 5 mA intensity for several seconds considered to be safe^55^. The brain-penetrating currents used in our studies remained well below these widely accepted values. One of 19 subjects in the ramp stimulation experiments (Fig. 5) requested to terminate ISP stimulation because of feeling dizzy. For the experiments shown in Fig. 6, we recruited 7 subjects (3 subjects overlapped with the experiments shown in Fig. 5 but were tested several weeks apart). In one of them, the instability of the electrodes was only discovered after the experiments and the results from this subject could not be analyzed due to excessive artifacts.

### Stimulation methods

Stimulating sponge electrodes for ISP were prepared from a 2 × 3 × 1.5 cm sponge glued to a 2 × 3 cm copper mesh, and glued to a rubber washer with the sponges inside, keeping approximately 2.5 cm distance between sponges. The rubber washer with the 12 electrodes was soaked in 0.9% saline solution and tightened gently around the head. Conductivity was further improved by putting electrode gel (SuperVisc, EasyCap GmBH, Germany) between the wet sponges and the skin. For abdominal ISP stimulation, the same sponge electrodes were placed around the trunk.

### Modeling of current-induced fields

To model the effect of soft-tissue resistivity on intracerebral electric fields, a finite element method model was constructed, and the theoretical values of the electric field inside the brain were calculated for different conditions using Comsol Multiphysics (Comsol, Burlington, MA, USA). Concentric spheres simulated the scalp, skull, cerebrospinal fluid, and brain (Supplementary Figure 9b). Conductivity values were set to 0.465, 0.015, 1.65, 0.3 s/m, respectively^17,35^. The dimensions of each layer were set to match one of the cadaver’s anthropometric data (8, 5, and 2 mm thicknesses for the skin, skull, and cerebrospinal fluid, respectively, while the brain diameter was set to 142.6 mm). Two virtual stimulating electrodes attached to each side of the head were modeled as conductors of 1 cm^2^ with the conductivity of copper (5×10^7^ s/m). Induced electric fields were calculated inside the model brain with a virtual 5 × 6 electrodes array, mimicking the experimental setup. Maxwell’s equations were solved within an adaptive mesh of 366619 elements, using a linear solver and a relative tolerance of 1e−6. The effect of skin and soft tissue resistivity change on electric fields was similar to those reported in earlier publications investigating postmortem resistance changes of soft tissues and muscle^37,38^.

We used a leaky-integrate and fire neuron model to visualize the ISP principle. Extracellular electric fields were derived from in vivo tACS measurements using 1 kHz sinusoid stimuli in the same arrangement as shown on Fig. 1a, but using epidural stimulation with screw electrodes. The directionless electric field intensities (35 mV/mm peak intensity) at each point were converted to intracellularly injected current values by multiplying with a factor (4.5 nA/mV/mm) to mimic transmembrane currents. A dimensionless leaky-integrate and fire neuron model was established in Matlab based on ref. ^31^. Parameters were set as the following: temporal constant of the membrane = 10 ms; resting membrane potential = −70 mV; membrane resistance = 1 MΩ; spiking threshold = −54 mV; spike peak potential = 20 mV; repolarization level = −80 mV. Extracellular electric field duration = 0.5 ms. The effects of three different magnitude current injections on the firing rate were demonstrated by the leaky-integrate and fire neuron model is illustrated in Fig. 2a.

### Electronic circuit of ISP stimulation

For the ISP stimulation approach, both positive and negative leads of the stimulus generators were connected to 12–12 TLP52-4 phototransistors (Toshiba, Japan). Bidirectional, ground-independent conductivity was achieved the following way. Two phototransistors were serially coupled through their emitter and collector, and the input signal from the waveform generator was fed into both the emitter and the collector end of the transistor doublet, through two Schottky-diodes, which allowed current flow only to the appropriate member of the doublet, depending on the polarity of the signal. The common segment of diode, the doublet, was connected to a stimulation electrode on the head. The same circuit was constructed for the other pole of the signal as well. Common driver signal to the infrared emitting diode sides opened all four transistors, but due to the diodes two of them were always floating, while the other two closed the circuit through the head (Supplementary Figure 1a). Six such circuits were used for the six electrode pairs, forming six quadruplets (blocks) of transistors. In rats, only three pairs were used. Blocks were activated in a pseudorandom order by transistor–transistor logic (TTL) pulses generated by a CD74HC4017 Decade counter (Texas Instruments, USA), driven by a 100-kHz TTL generator (ADG3051C, Tektronix, USA).

In sessions employing variable ISP intensities in human subjects, the phototransistors were replaced with ADG412 high-speed analog switches (Analog Devices, Norwood, MA, USA) and the control TTL signals were generated by a PIC18F4525 (Microchip, Chandler, AZ, USA) microcontroller and isolated by ADuM1400 (Analog Devices) digital isolators. This circuit allowed the unrestricted flexible assignment of stimulus polarities to the electrodes. This latter circuitry was also used for the experiments on rats when we compared the spatial effect of ISP and TES pulses.

### EEG recording during ISP stimulation

EEG scalp recordings were performed by a 16-channel V-Amp amplifier and ActiCap BP active electrodes (Brain Products GmBH, Germany). Impedances were measured online and adjusted to remain below 20 kΩ by applying electrode gel. Electrodes were placed according to the International 10/20 electrode scheme. The broad dynamic range of the active electrodes, and their buffering capacity allowed the low-noise transmission of EEG signals and stimulus artifacts without on-head amplification. To prevent the saturation of the amplifier, the output range of the active electrodes was matched to the input range of the EEG amplifier through custom-made voltage dividers.

### Data processing

The recorded data were analyzed by custom-written scripts in MATLAB (MathWorks, USA). Single unit analyses were performed on the time series of semi-automatically clustered and manually refined unit clusters of extracellular action potential waveforms, as described earlier (PHY^56,57^). Only well isolated single units were used in the analysis. Units were categorized as putative pyramidal cells or interneurons based on their physiological properties.

To measure the electric field in cadavers and rats, 500 sinus cycles were averaged for each condition and then the peak-to-peak amplitude was measured for each channel. The first spatial derivative of these voltage signals was calculated .

In the silicon probe experiments, we measured the impedance of all contact sites at 10, 100, and 1000 Hz (Intan recording software, Intan Technologies, Los Angeles) and excluded those channels whose impedance values were higher than 2 MOhm: 500 sinus cycles were averaged for each condition and then the peak-to-peak amplitude was measured for each channel and a mean shank voltage was computed. Finally, we calculated the first spatial derivative of these potential values.

### Statistical tests

Unless otherwise noted Student’s paired *t*-test with Bonferroni correction was used for pairwise data comparison, Pearson’s linear correlation was calculated for correlation analyses and mean ± SEM values are displayed with the full data sets superimposed. Data with non-normal distribution are reported as median and interquartile range (IQR). Boxplots with whiskers denote the medians, interquartile ranges, and full ranges. For data sets with non-normal distribution, non-parametric tests were used instead. Welch’s correction was applied when variances were not equal. To preserve visibility of the figure panels, significance levels of <0.05, <0.01, and <0.005 are marked by one, two, or three asterisks, respectively. For simplicity, *P* values smaller than 0.001 are reported as <0.001 instead of stating the absolute value. The detailed conditions, numeric results, and effect sizes of the statistical comparisons are listed in the Supplementary Results.

### Analysis of whole-cell patch clamp recordings

To remove the stimulation artifacts, after retracting the patch-pipettes, the artifacts of the same set of electrical stimuli applied during the whole-cell recordings were recorded extracellularly. The recorded artifacts were subtracted from the intracellularly recorded potentials to recover the true transmembrane potentials^25^. Power spectra of the stimulated and control epochs were calculated on a trial-by-trial basis, using fast Fourier transform, before averaging. Spectra were whitened by the 1/*f* method.

### Noise removal and analysis of EEG activity

The stimulus artifact was removed offline by subtracting a triggered moving average (*t* = 10 epochs), followed by triple-sweeps of 100th order zero phase-lag high-pass finite impulse response filter (*f* = 2 Hz) in MATLAB.

For analyses performed on the time domain (e.g., alpha amplitude) the artifact-free signal was filtered in the alpha band with a zero phase-lag fourth-order Butterworth filter. Instantaneous alpha amplitudes were determined by calculating the magnitude of the Hilbert-transformed filtered signal, and binned based on the corresponding ISP amplitude and phase. Binned values were averaged across epochs. To estimate the amplitude of the remaining electrical noise time locked to the epochs, signal was first averaged across epochs, and then Hilbert transformed. This approach preserved time-locked features. For frequency domain analyses, spectral amplitudes were calculated using fast-Fourier transformation, and smoothed using a moving average filter (width = 2 Hz). 120–140 Hz was chosen as a control frequency band, as this range does not represent measurable physiological oscillatory signals on the scalp but would still mirror the presence of broadband electrical artifacts. For time-resolved spectral analysis, spectra were calculated using a multitaper fast Fourier transform on 1-s long consecutive segments. Spectra were whitened by multiplying each frequency by the frequency value (1/*f* method).

### Frequency-amplitude and phase-amplitude analysis of EEG

We employed two complementary analyses to assess the modulation of EEG amplitude by the phase of the sinusoidal ISP stimulation current. Analyses were performed on 1-min-long consecutive epochs, and the epoch results were pooled. First, we applied the complex wavelet transform using Morlet mother wavelets to calculate the amplitude and phase for a wide range of EEG frequencies. Wavelet amplitudes were calculated from 1 to 30 Hz at 59 levels from the artifact-free EEG and wavelet phase for 21 levels from 0.5 to 5 Hz at 15 levels from either the original EEG or a synthetic signal constructed from the stimulation pulses. Phase–amplitude cross-frequency coupling was quantified using a modulation index (MI^58^). To quantify frequency–amplitude modulation, 2-D comodulograms were constructed with the MI values for every phase–amplitude frequency pair and the maximal MI in the band of interest was detected^59^. For phase–amplitude modulation, phase time-series were binned into phase intervals and the mean wavelet amplitude was calculated for each of them and *z*-scored. Phase time-series were binned into phase intervals and the mean wavelet amplitude was calculated for each of them^59^.

For a complementary phase–amplitude analysis performed on the time domain, the estimated peak-to-peak amplitude values of the individual alpha waves were binned based on the actual stimulus phase, and alpha amplitude values during the stimulus peak and trough bins (45, 90, 135, 225, 270, 315°) were compared to the alpha amplitude values present at the transitional phase (0° and 180°) bins using a *t*-test.

### Data availability

The data sets generated and analyzed during the current study are available upon reasonable request from the corresponding authors for further analyses.

## Electronic supplementary material

Supplementary Information
