# Supplementary material for: Direct effects of transcranial electric stimulation on brain circuits in rats and humans
Source: Nat Commun. 2018 Feb 2;9:483. doi: 10.1038/s41467-018-02928-3 (PMC5797140; doi:10.1038/s41467-018-02928-3)
Supplement: Supplementary file 1 — Supplementary Information [file 41467_2018_2928_MOESM1_ESM.pdf]

Supplementary Information

Direct effects of transcranial electric stimulation on brain circuits in rats and humans

Mihály Vöröslakos[1,4], Yuichi Takeuchi[1], Kitti Brinyiczki[2], Tamás Zombori[2], Azahara Oliva[1,4], Antonio Fernández-Ruiz[1,4], Gábor Kozák[1], Zsigmond T. Kincses[3], Béla Iványi[2], György Buzsáki[4,5,6], Antal Berényi[1,4]

[1]MTA-SZTE "Momentum" Oscillatory Neuronal Networks Research Group, Department of Physiology, University of Szeged, Szeged, Hungary
[2]Department of Pathology, University of Szeged, Szeged, Hungary
[3]Department of Neurology, University of Szeged, Szeged, Hungary
[4]The Neuroscience Institute, [5]Department of Neurology and [6]Center for Neural Science, New York University, New York, New York 10016

#	Age	Sex	Weight (kg)	Post-mortem age (day)	Brain water (%)	Brain weight (g)	Skull circumference (cm)	Skull thickness (mm)								Cause of death
								L1	L2	L3	L4	R1	R2	R3	R4	
**1/18**	94	M	43	3	80.37	1235	50.7	6	7	5	6	5	5	4	6	Bronchopneumonia
**1/19**	86	M	59	6	81.56	1360	52.3	4	4	4	3	3	5	4	4	Bronchopneumonia
**1/20**	87	F	67	8	78.29	1115	49	6	6	5	4	5	4	4	5	Klatschkin-tumor
**1/21**	92	F	54	7	81.01	1075	49.8	8	6	5	5	9	4	6	4	Heart failure
**2/2**	94	M	58	5	82.57	1105	47.8	6	5	4	5	6	6	4	4	Heart failure
**2/3**	65	M	57	5	82.94	1025	-	-	-	-	-	-	-	-	-	Acute myeloid leukemia
**2/4**	67	M	53	7	80.49	1340	50.8	4	4	4	5	3	5	4	6	Lung adenocarcinoma
**3/2**	82	F	51	4	78.95	1200	49.5	4	3	5	6	4	4	5	6	Pancreas adenocarcinoma
**3/3**	76	F	78	7	83.15	1210	52	9	6	4	6	9	5	5	6	Pulmonary embolism
**3/4**	94	F	45	3	83.87	1105	49.2	10	4	4	6	3	5	5	3	Bronchopneumonia
**3/5**	88	F	62	3	81.73	1220	50.5	10	5	6	4	3	4	4	5	Bronchopneumonia
**3/6**	69	F	43	3	86.36	1225	-	-	-	-	-	-	-	-	-	Bronchopneumonia
**3/s1**	80	F	120	6	83.33	1210	49.6	6	5	3	4					Invasive ductal adenocarcinoma of the breast
**3/s2**	59	F	78	7	-	1120	50.4	6	4	4	5					Heart failure
**4/1**	82	M	-	3	88.77	1455	54	-	5	-	-	-	4	-	-	Hypovolemic shock
**4/2**	73	M	-	7	81.54	1255	51.8	-	5	-	-	-	5	-	-	Bronchopneumonia
**4/3**	80	M	-	2	87	1480	54.2	-	4	-	-	-	4	-	-	Acute myocardial infarction
**Mean**	**80.5**		**62**	**5.1**	**82.6**	**1219.7**	**50.8**	**6.6**	**4.9**	**4.4**	**4.9**	**5.0**	**4.6**	**4.5**	**4.9**	

Supplementary Table 1. **Anthropometric data of the cadavers**.

L1-4 and R1-4 refer to the 4 location of stimulating electrodes on the left and right sides, respectively. L1 and R1 denote the two most frontal locations.

Analysis	Figure	1/18	1/19	1/20	1/21	2/2	2/3	2/4	3/2	3/3	3/4	3/5	3/6	3/s1	3/s2	4/1	4/2	4/3
**Stimulus intensity**	Figure 4e	x	x	x	x													
**Stimulus frequency**	Figure 4f								x	x	x	x	x					
**Electrode size**	Figure 4g								x	x								
**Voltage-current relationship**	Figure 5b	x	x	x	x	x	x	x	x	x			x					
**Transcutaneous vs. subcutaneous**	Figure 5c-f		x			x	x	x	x	x			x			x	x	x
**Subcutaneous vs. Epidural stim.**	Figure 5e-f										x	x	x					
**Skull thickness**	Figure 5g	x	x	x	x				x	x	x	x						
**DC vs. AC stimulation**	Supp. Fig. 4			x	x													
**Coronal plane**	Supp. Fig. 5					x												
**Stimulation mode**	Supp. Fig. 6a					x												
**Phase shift**	Supp. Fig. 6c	x		x	x													
**Fronto-lateral arrangement**	Supp. Fig. 7													x	x			
**Skull thickness**	Supp. Fig. 8	x	x	x	x				x	x	x	x						

Supplementary Table 2. **Overview of the experiments and analyses performed on individual cadavers**

Figure	Stimulation waveform	Intensity	Frequency	Stimulation electrode surface (mm2)	Number of stimulation electrode pairs	Targeted hemisphere	Remarks
**Figure 1a**	Sine	3 V	1000	4	3	NA	
**Figure 1b (example)**	Sine	3V	1000	4	1	NA	
**Figure 1b (population)**	Sine	3V	10,20, 50, 100, 200, 500, 1000, 2000	4	1	NA	
**Figure 1d, e**	Sine	10, 20, 50, 100, 200 µA	10, 100, 1000	4	1	NA	
**Figure 2**	DC	± 100, 200, 400, 600, 800 µA	NA	4	1	NA	(1)
**Figure 3**	ISP	5V, 200 µA	NA	3.6	3	Left or right	(2)
**Figure 4d**	Sine	5V	100	78	4	NA	
**Figure 4e**	Sine	1, 2, 3, 4, 5V	200	78	1	NA	
**Figure 4f**	Sine	5V	5, 10, 20, 50, 100, 200, 500, 1000, 2000,	78	1	NA	
**Figure 4g**	Sine	5V	1000	78	6	NA	
**Figure 5c**	Sine	5V	200	78	4	NA	(3)
**Figure 5d**	Sine				2	NA	(3)
**Figure 6, 7g**	ISP	1.5, 3, 4.5, 6, 7.5 mA	1	200	6	Midline	(4)
**Figure 7a-f**	ISP	2, 4.5, 7, 9 mA	1	200	6	Midline	
**Figure 8**	ISP	2, 6 mA	1	200	6	Midline / N/A	
**Supp. Fig. 1b**	ISP	7 mA	1	200	6	Midline	
**Supp. Fig. 1c**	ISP	2, 9 mA	1	200	6	Midline	
**Supp. Fig. 4**	DC / Sine	5V	NA / 200	78	1	NA	
**Supp. Fig. 5**	Sine	5V	1000	78	4	NA	
**Supp. Fig. 6a**	Sine	1, 3, 5V / 50, 100, 150 µA	1000	78	1	NA	
**Supp. Fig. 6c**	Sine	5V	100	78	2	NA	
**Supp. Fig. 7**	Sine	4V	10, 20, 100, 200, 1000	78	3	NA	
**Supp. Fig. 8**	Sine	5V	100	78	1	NA	
**Supp. Fig. 9d**	Sine	1V	100	4	1	NA	
**Supp. Fig. 10**	ISP	1.5, 3, 4.5, 6, 7.5 mA	1	200	6	Midline	

Supplementary Table 3. **Overview of stimulation parameters for each experiment and figure.**
Notes below explain the rationale for the choice of stimulation parameters in different experiments.

(1) In contrast to the human measurements, neuron stability and brain state changes limited the duration of the experiment available for rodent data collection. In the intracellular experiments, we chose DC stimulation (right side was the cathode) because artifact issues are easier to deal with DC stimulation (only onset and offset artifacts had to be removed). In experiments where sine waves were used, the positive half of the sine wave corresponded to left-anodal stimulation, while the negative half to the right-anode.

(2) Each trial consisted of 3 x 2.5 µs pulses repeated at 133 kHz (100% duty cycle) for 500 ms and followed by 1 s pause (see also Supplementary Figure 2a).

(3) To achieve high signal-to-noise ratio in the cadaver experiments, we first identified the strongest stimulation intensity, which did not saturate the amplifier. This variability across brains should not affect our results since we demonstrate a nearly perfect linear correlation between with the applied intensity and induced fields (Figure 4). In a subset of experiments, we used the same intensity (1 mA) for scalp, skull and brain surface stimulation (Figure 5).

(4) In the human ISP experiments, 6 x 10 µs pulses were repeated at 16.66 kHz (100% duty cycle). The amplitude of the pulses was modulated by a 1-Hz sine wave, linearly ramping up from zero to maximum in 6 seconds, then ramping down to zero in 6 seconds (see also Supplementary Figure 2b).

Subjects	Pain**		Phosphenes		Dizziness		Metallic taste	
	ISP>Shuffled	Shuffled > ISP	ISP (6mA)	Shuffled (6mA)	ISP (6mA)	Shuffled (6mA)	ISP (6mA)	Shuffled (6mA)
III/1	No	Yes	Yes	No	Mild	No	Yes	No
III/2	No	Yes	Yes	No	Mild	No	Yes	No
III/3	No	Yes	Yes	No	Mild	No	Yes	No
III/4	No	Yes	Yes	No	Mild	No	Yes	No
III/5	No	Yes	Yes	No	Mild	No	Yes	No
III/6	No	Yes	Yes	No	Mild	No	Yes	No
III/7*	No	Yes	Yes	No	Mild	No	Yes	No
III/2 (eyes open)	No	Yes	Yes	No	Moderate	No	Yes	No
III/4 (eyes open)	No	Yes	Yes	No	Strong	No	Yes	No
III/6*** (eyes open)	No	Yes	Yes	No	Strong	No	Yes	No

Supplementary Table 4. **Comparison of subjective report of 'ISP' and 'Shuffled ISP' stimulation-induced effects in human subjects**.
After each 5-min stimulation epoch, the subjects were asked: did you see 'sparks'? Did you feel dizzy? Did you feel taste in your mouth? What was it like? Subjects were not asked to give a magnitude but they spontaneously reported mild, moderate and strong dizziness.

In three subjects (III/2, III/4 and III/6 – eyes open sessions) the protocol was repeated while they were asked to keep the eyes open for both the stimulation and control periods. These sessions were used to further estimate the severity of dizziness during ISP and Shuffled ISP protocols only, and were not included in the analysis of EEG. Note that all three subjects reported more severe dizziness compared to the eyes closed sessions, and in one occasion an altered peripheral vision. These side effects were completely absent when the Shuffled ISP protocol was applied.

* This subject was excluded from the analysis due to excessive electrical artefacts.

**At the termination of both ISP and Shuffled stimulations the subjects were asked: Which epoch was more unpleasant, the first or the second?

*** This subject reported altered peripheral vision at 2 mA ISP but not at 7 mA.

Subjective discomfort varied from mild sensation to burning feeling of the scalp but its magnitude was not quantified.

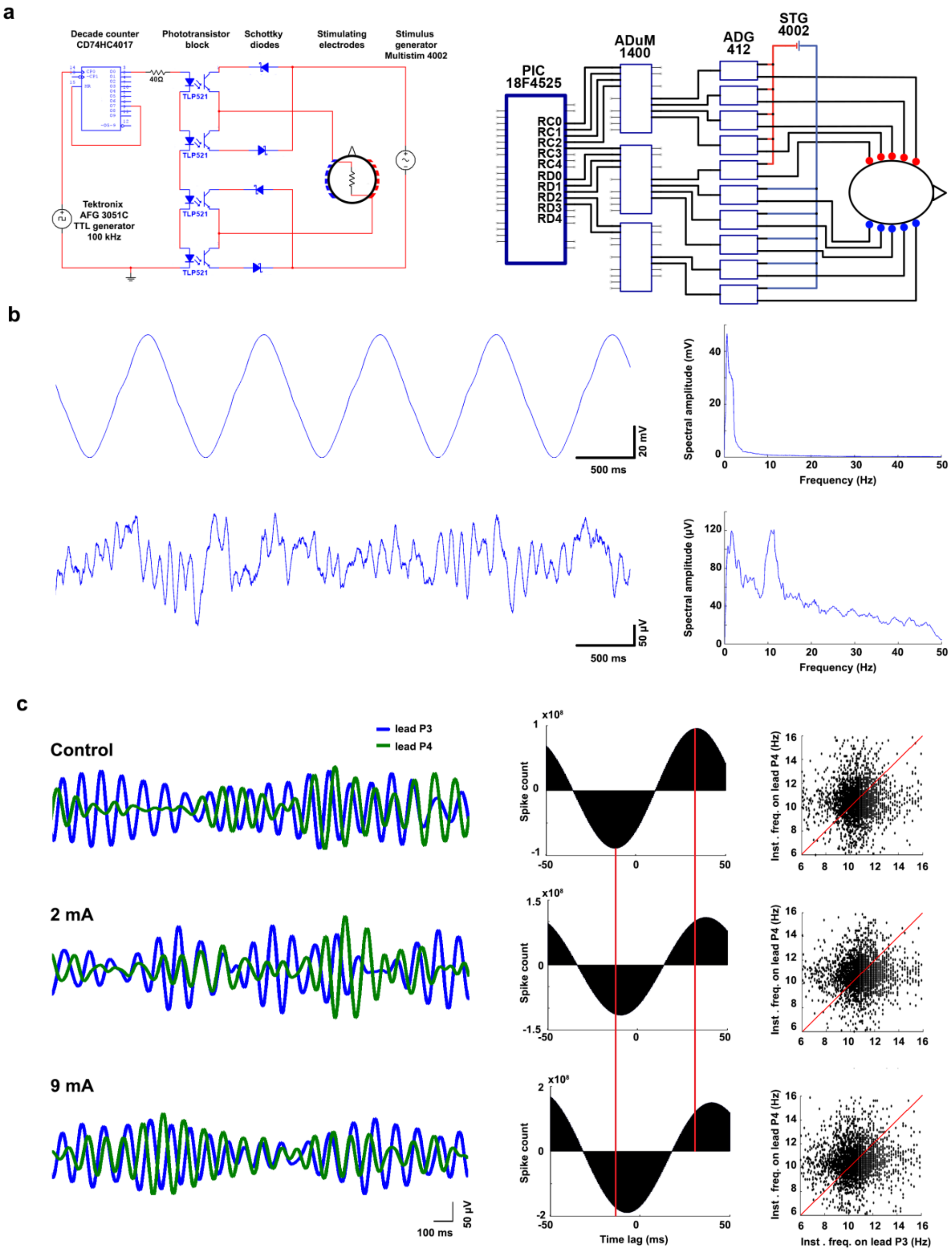

Supplementary Figure 1. **Circuit schematics of ISP Stimulator and artefact removal.**

(a) Left: Schematics of fast-pulse ground-independent signal-splitter circuit for one electrode pair. Driver TTL signal is generated by an external pulse generator, which is advancing a decade counter. Counter is driving six identical bipolar switching modules, each built of four phototransistors. Right: An alternative solution used a microcontroller (Microchip PIC18F4525) and digital isolators (ADuM1400) that allow more flexibility of stimulation patterns. Ground-independent switching is performed by high-speed analog switches (ADG412) instead of phototransistors. **(b)** EEG traces during ISP stimulation. Example trace showing EEG recording before (top left trace) and after artefact removal (bottom trace). Right panels: corresponding power spectra of EEG traces shown on the left. Stimulus intensity = 7 mA. **(c)** Alpha-band filtered EEG signals recorded by the left and right

occipital leads (left panels). Note that the phase and amplitude of alpha waves in the two hemispheres vary relatively independently from each other under both control and ISP stimulation (2 mA and 9 mA) conditions, implying that the traces are free of common electrical artefacts. Time-lag of cross-correlogram peaks is also similar under control and ISP stimulation conditions (red vertical bars denote correlogram peak and trough of the 0 mA condition for better visibility). Instantaneous frequencies of the EEG traces from the two hemispheres vary from event to event (Pearson's linear correlation; R= -0.0024, 0.01 and -0.004; P = 0.89, 0.57, 0.82; n=3053, 3092 and 3098 from a single subject, at 0, 2 and 9 mA intensities, respectively). Note that stimulation-induced artefacts are expected to have constant phase and amplitude ratios at all recording positions. Full data distributions are shown on the scatter plots.

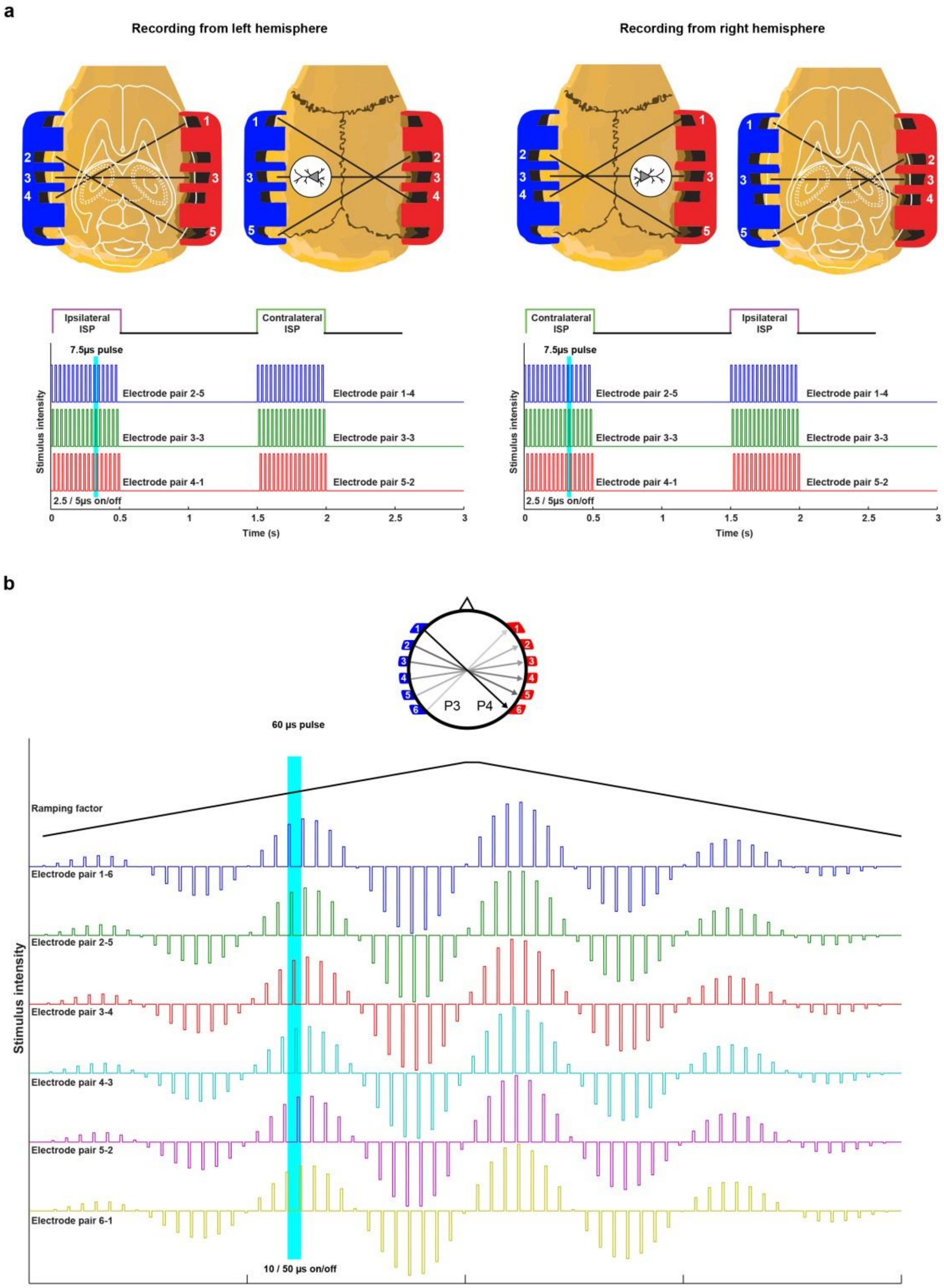

Supplementary Figure 2. **Illustration of ISP protocol.**

(a) Upper part shows the schematics of the recording and stimulating setup in rodent experiments. Neuronal activity was recorded from both hemispheres simultaneously (white circles corresponds the location of craniotomies). The ISP was alternatingly focused to left or right hemisphere in an interwoven fashion, so that neurons in the left (or right) hemisphere were more strongly modulated by ISP focused to the left (or right) hemisphere. Spiking activity of some neurons contralateral to the focused hemisphere was suppressed, possibly because of the opposite geometric orientation of neurons compared to the ipsilateral hemisphere (note the different orientation of the schematic

neuron in the white circle). Lower part shows the schematics of the stimulation sequence for two consecutive trials. Each trial consisted of 3 x 2.5 µs pulses repeated at 133 kHz (100% duty cycle) for 500 ms and followed by 1 s pause (Supplementary Table 3). Note that the duration of the 2.5 µs pulses are shown disproportionally longer for better visibility. **(b)** Upper part shows the position of the recording (P3 and P4) and stimulating electrodes in human measurements. Lower part shows a single trial, which consisted of 6 x 10 µs pulses repeated at 16.66 kHz (100% duty cycle). The amplitude of the pulses was modulated by a 1-Hz sine wave, linearly ramping up from zero to maximum in 6 seconds, then ramping down to zero in 6 seconds. Please note that the length of the 10 µs pulses and the ramping time are shown disproportionally for better visibility.

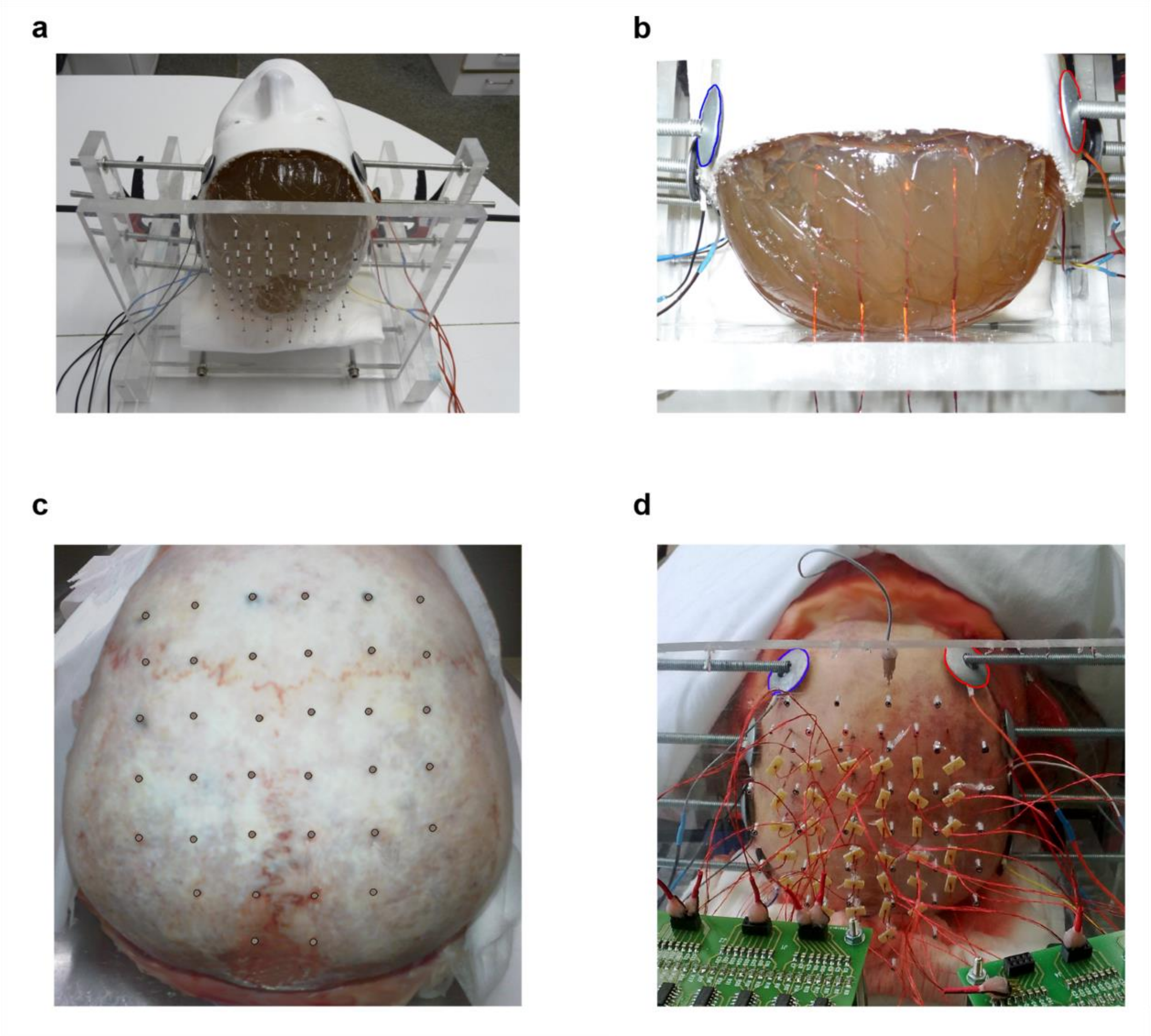

Supplementary Figure 3. **Photographs of cadaver recording arrangements**.
Supporting plexiglass frame with pre-drilled holes for electrode positioning **(a)**, and recording electrode penetration in a jelly mannequin brain **(b)**. **(c)** Locations of holes drilled to introduce recording electrodes (n = 36). **(d)** Set up for intracranial voltage measurements with transcranial stimulation. Stimulation electrodes are marked with red and blue circles.

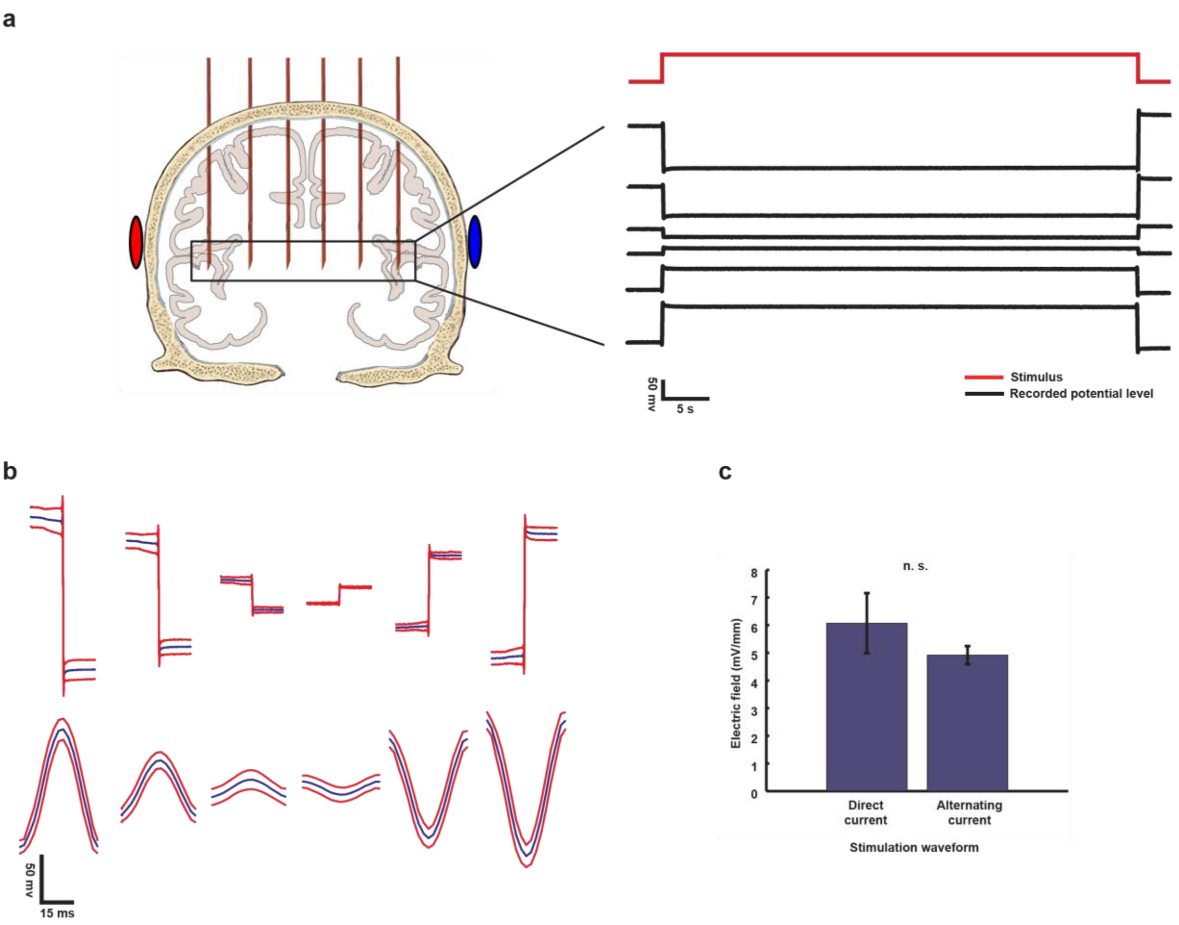

Supplementary Figure 4. **TES generates constant electric fields over time**.
(a) Schematic drawing of the location of six recording electrode sites spanning the extent of the brain and the stimulation electrode pair. Representative traces of voltage signals recorded on the six contact sites (black) and the delivered stimulus waveform (red). Long (50 s) DC pulses and measured voltage changes indicate ohmic properties of the tissues **(b)** Comparison of tDCS and 20 Hz tACS using identical currents resulted in quantitatively similar intracerebral peak potential values on the six recording sites (n = 40 repetitions in 2 cadavers, 5V, mean ± 2 SD). The effect of tDCS was recorded using Ag/AgCl electrodes to prevent electrode polarization. tACS response was recorded with metal (Nicrothal) electrodes. Stimuli were delivered through Ag/AgCl electrodes in both cases. **(c)** Mean electric fields were similar for both tDCS and tACS application (Stimulus intensity = 5V, P = 0.12, Mann-Whitney U-test, N = 10 from two cadavers).

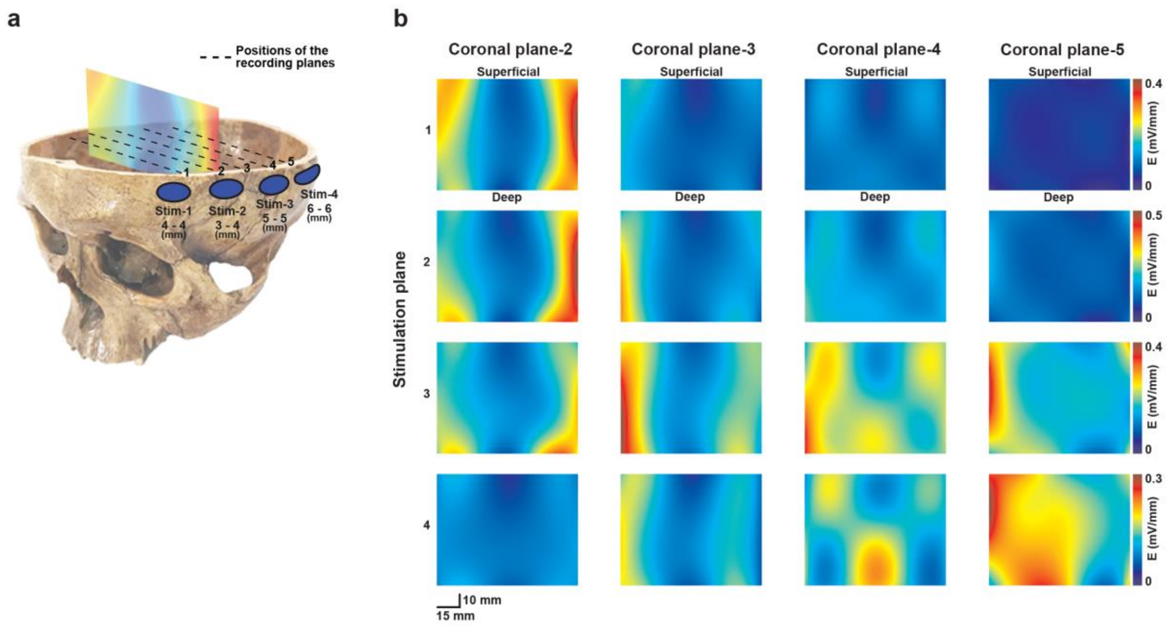

Supplementary Figure 5. **Intracerebral electric fields are affected by stimulation electrode locations.**

(a) Schematic drawing of recording planes (dashed lines) and the positions of the stimulating electrode pairs. Numbers below the ellipsoids indicate the skull thickness at the stimulation electrodes. **(b)** Largest gradients did appear in the coronal planes of the stimulating electrodes. Note different color calibrations for different maps. The left versus right asymmetry and the different magnitude fields may be explained by the different thickness of the skull at the various coronal planes.

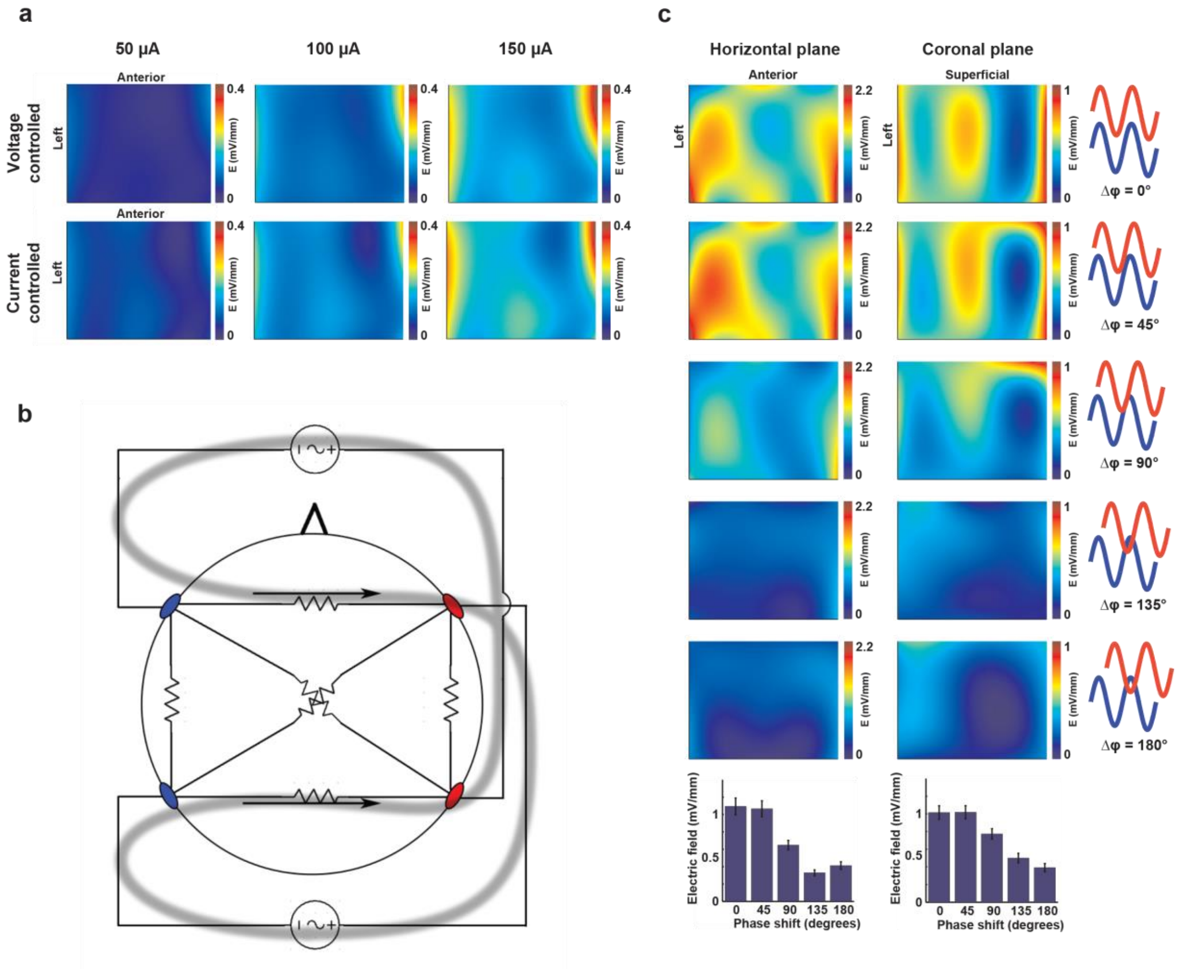

Supplementary Figure 6. **Multiple simultaneous stimulator pairs do not focus the intracerebral electric gradients**.

(a) Current and voltage mode stimulations result in identical effects. Single session example shows distribution maps with voltage mode (upper row, 1, 3 and 5 V from left to right, respectively), and current mode (lower row, 50, 100 and 150 µA intensities from left to right, respectively). Intensities for current mode stimulation were chosen to match the calculated current intensity in voltage mode stimulation sessions (see Materials and Methods). Note identical distribution maps. **(b)** Equivalent circuit schematic for the application of multiple independent stimulating pairs in an intersectional arrangement, resembling gamma-ray radiosurgery. Note that due to the common conductive medium, the currents from the two stimulators couple serially, mimicking the effect of one large surface electrode pair and/or increased stimulus intensity, but they don't reach spatial selectivity **(c)** As predicted from the model on panel **b**, shifting the relative phase of the sinusoidal stimuli from two independent stimulator pairs reduce the induced field in both the horizontal and coronal planes. Bottom graphs: peak voltage gradient values as a function of phase shift both in the horizontal (left column, n = 60 gradient values in 3 cadavers) and coronal plane (right column, n = 60 gradient values in 3 cadavers).

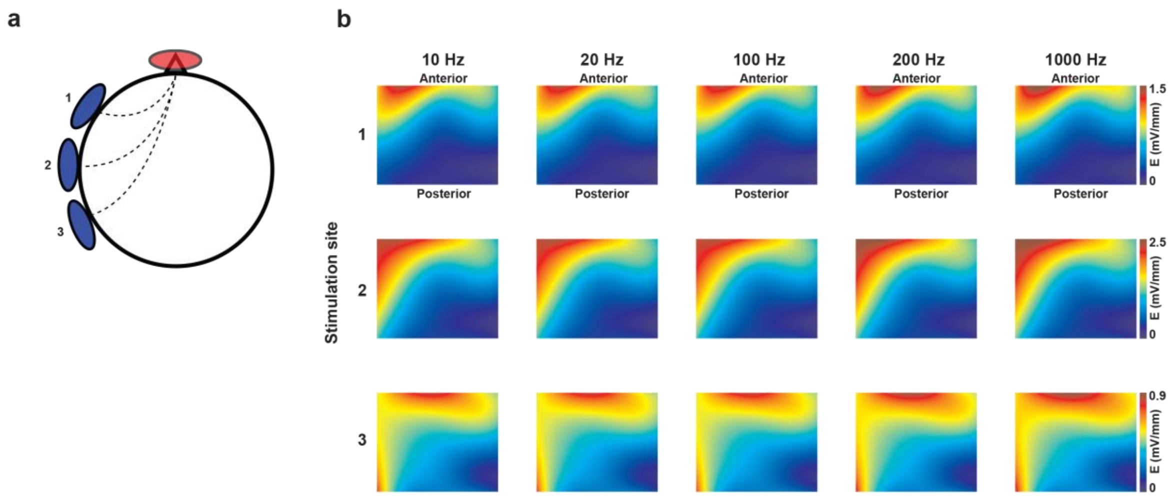

Supplementary Figure 7. **The effect of electrode arrangement on the spatial distribution of induced field**.

(a) Schematic of transcranial electrode locations in a cadaver. Red ellipse, cathode placed on the forehead in the sagittal plane; blue ellipses, positions of three anode locations. **(b)** Intracerebral voltage gradient maps; each row corresponds to one anodal location. Note the frequency independence (columns) of the gradient maps. Different inter-electrode distances induced variable size and shape of field distributions.

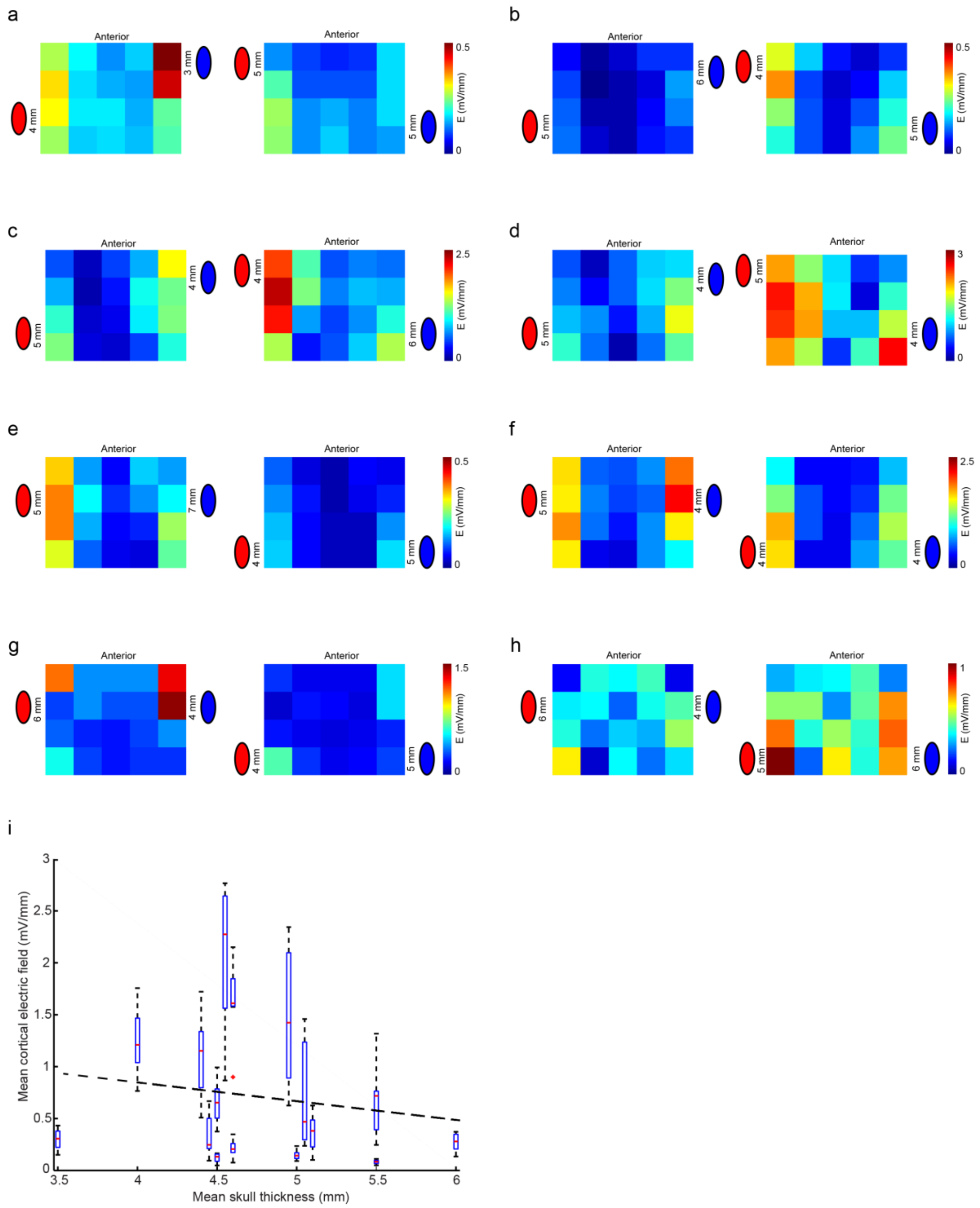

Supplementary Figure 8. **Effect of skull thickness on the electric field magnitude and distribution.** **(a - h)** Intracerebral voltage gradient maps (horizontal plane) of #3/2 – #3/5 and #1/18 – #1/21 cadavers. Cathode (red ellipse) and anode (blue ellipse) were placed bilaterally on the skull. The numbers below the ellipses indicate the skull thickness under the stimulating electrode. Different stimulation electrode locations and/or skull thicknesses induced electric field distributions with variable size and shape in the brain. Note the different color calibrations for the different cadavers (a – h). **(i)** Median and interquartile range of the mean cortical electric field (four 'cortical' values on each of the two sides) as a function of mean skull thickness for two conditions in each cadaver (n = 8). Increasing skull thickness is decreasing the magnitude of electric field in the brain (Pearson's linear correlation; R = -0.2332; $P < 0.05$; n = 128 cortical electric field intensities from 8 cadavers,).

a

#	Post mortem age (day)	Brain water %
2013-18	3	80.37
2013-19	6	81.56
2013-20	8	78.29
2013-21	7	81.01
2014-2	5	82.57
2014-3	5	82.94
2014-4	7	80.49
2015-2	4	78.95
2015-3	7	83.15
2015-6	3	86.36
2015-spring-1	6	83.33
2015-spring-2	7	-
Alive		77-78

b

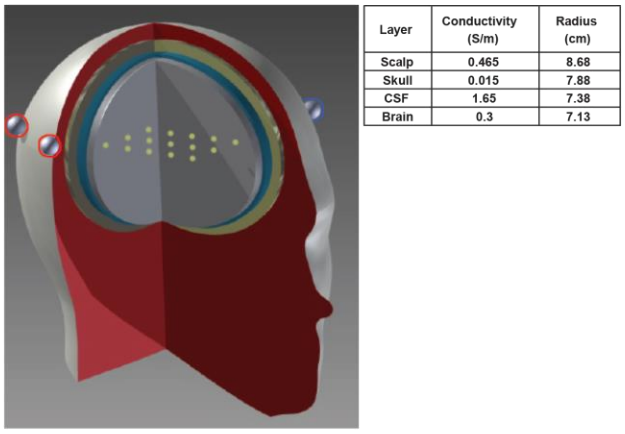

Layer	Conductivity (S/m)	Radius (cm)
Scalp	0.465	8.68
Skull	0.015	7.88
CSF	1.65	7.38
Brain	0.3	7.13

c

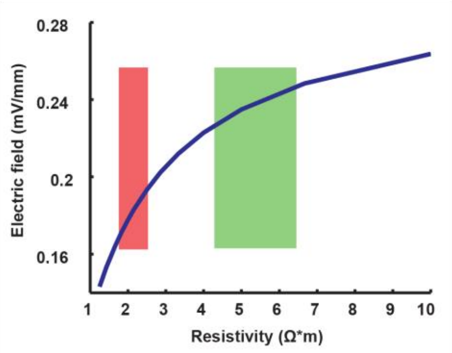

d

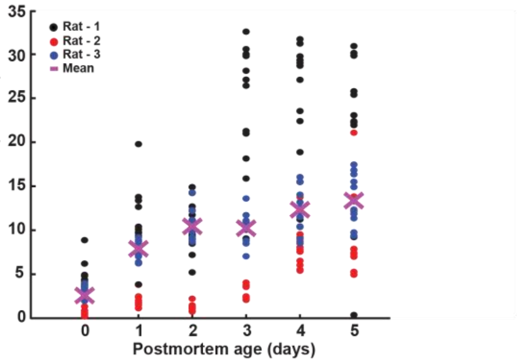

Supplementary Figure 9. **Comparison of cadaver and in vivo conditions.**
(a) Water content of brain specimens did not change significantly over post mortem days, indicating that the cadaver brains did not undergo significant desiccation. For comparison, literature value for in vivo hydration level is shown, too ('Alive'). **(b)** Schematics and parameters of the finite element model used for simulations. Positions of the virtual recording electrodes were arranged to match the cadaver recording locations. Conductivity and radius values for the concentric geometries are shown in the table on the right. **(c)** Resistivity values of the skin layer determine the strength of intracerebral gradients generated by a given stimulus intensity. Note that larger skin resistances (as in cadavers) result in larger intracerebral effect due to the smaller shunting effect of the skin. Green and orange boxes denote the range of live and post-mortem skin resistances, respectively, based on literature data. **(d)** Comparison of *in vivo* and *postmortem* conditions in rats. Induced voltage gradients in vivo and 1 to 5 days after death (12 recording sites, seven stimulus frequencies in 3 rats). The increased voltage gradients after death likely reflect the reduced shunting properties of the drying postmortem scalp.

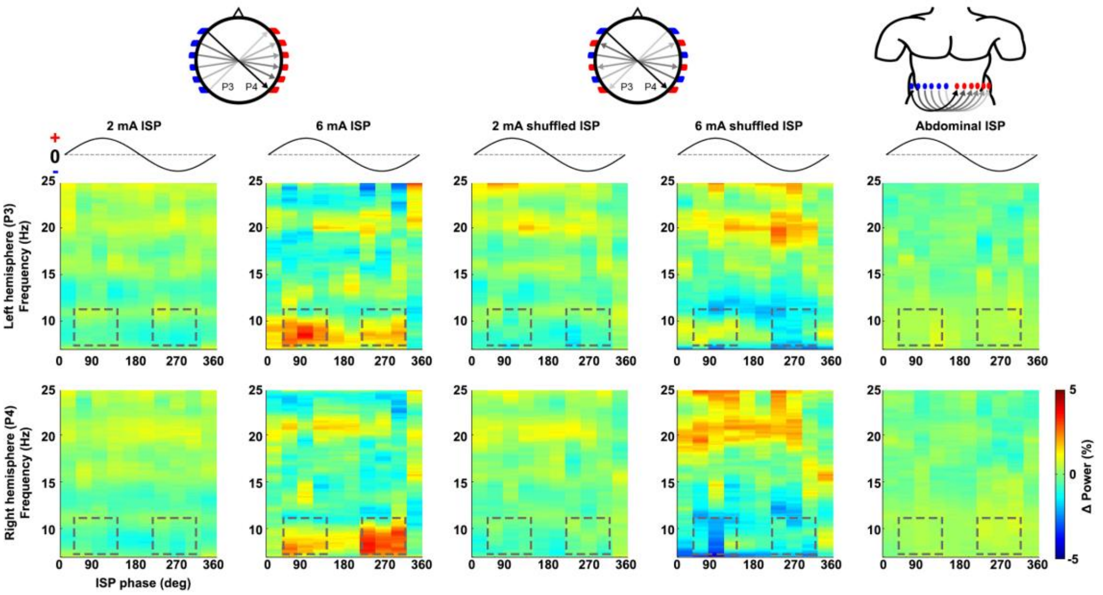

Supplementary Figure 10. **ISP stimulation-induced modulation of EEG.**

Panels represent ISP stimulus-induced phase-modulation of power in different frequency bands. Alpha power band is highlighted by squares. Each panel shows the difference between stimulation (5 min) and the preceding nonstimulated 1-min long control periods (as shown in Figure 8a). Note that anodal stimulation of the respective hemisphere at 6 mA ISP strongly increases alpha power, while shuffled ISP stimulation is much less effective (for quantification and statistics, see also Figure 8b). Power in the beta band is also increased by stimulation. Based on n = 6 subjects. Abdominal wall stimulation (6 mA ISP) had no effect (n = 2 subjects).

Supplementary Results - Statistics Table

Figure	Panel	Test used	n		Descriptive statistics shown	P value	Degree of freedom & F/t/z/R/etc values
			Exact value	Definition			
1	**b** top	Mann and Whitney U test	20	Modulated area from 4 rats	Box plots with whiskers denote medians, interquartile ranges and full ranges	P = 0.0409	Z = 2.0443
	**b** bottom	Mann and Whitney U test	20	Modulated area from 4 rats		P = 0.0187	Z = 2.3510
	**e**	Paired t-test	20	5 intensities from 4 rats		P < 0.001	t(19) = -19.5773
2	**a** transcutaneous	Pearson's linear correlation	13	Transmembrane potential change of 8 neurons with 13 intensities	Full dataset + error bars are mean ± SEM	P = 0.002	R = 0.8626
	**a** subcutaneous	Pearson's linear correlation	13	Transmembrane potential change of 9 neurons with 13 intensities		P < 0.001	R = 0.9725
	**a** subcutaneous	Paired t-test with Bonferroni correction for multiple comparison	25	Membrane potential difference values from 5 neurons in 4 rats		P = 0.003, 0.004, 0.046, 0.14, 1.60, <0.001, <0.001, <0.001, for -800,-600,-400,-200, 200, 400, 600, 800 µA all vs 0 µA	t(24) = -4.61, -4.53, -3.45, -2.88, 1.58, 7.25, 7.35, 6.74, for -800,-600,-400,-200, 200, 400, 600, 800 µA all vs 0 µA
	**a** transcutaneous	Paired t-test with Bonferroni correction for multiple comparison	40	Membrane potential difference values from 8 neurons in 3 rats		P = 2.62, 2.57, 0.015, 0.091, 2.62, 0.044, 0.008, 0.003, for -800,-600,-400,-200, 200, 400, 600, 800 µA all vs 0 µA	t(39) = -1.06, -1.15, -3.74, -2.99, 1.07, 3.35, 3.98, 4.37, for -800,-600,-400,-200, 200, 400, 600, 800 µA all vs 0 µA

	**b** transcutaneous	Pearson's linear correlation	13	Mean firing rate of 8 neurons with 13 intensities		P = 0.0075	R = 0.7956
	**b** subcutaneous	Pearson's linear correlation	13	Mean firing rate of 9 neurons with 13 intensities		P < 0.001	R = 0.9542
	**b** subcutaneous	Paired t-test with Bonferroni correction for multiple comparison	25	Firing rate difference values from 5 neurons in 4 rats		P = 0.044, 0.028, 0.153, 0.33, 0.001, 0.065, <0.001, <0.001, for -800,-600,-400,-200, 200, 400, 600, 800 µA all vs 0 µA	t(24) = -3.49, -3.69, -2.84, -2.45, 5.02, 3.27, 6.08, 5.54, for -800,-600,-400,-200, 200, 400, 600, 800 µA all vs 0 µA
	**b** transcutaneous	Paired t-test with Bonferroni correction for multiple comparison	35	Firing rate difference values from 7 neurons in 3 rats		P = 4.61, 4.42, 4.61, 1.60, 1.64, 0.15, 0.39, 0.046, for -800,-600,-400,-200, 200, 400, 600, 800 µA all vs 0 µA	t(34) = 0.34, 0.16, -0.44, 1.58, 1.48, 2.74, 2.29, 3.31, for -800,-600,-400,-200, 200, 400, 600, 800 µA all vs 0 µA
	**c**	Mann-Whitney U-test with Bonferroni correction for multiple comparison	30	x 150 spectral power values for all nine conditions	Error bars are mean ± SEM	Frequency bins significantly different from the control condition (p<0.05) are marked on the figure	Not shown
	**d**	Mann-Whitney U-test with Bonferroni correction for multiple comparison	35	x 150 spectral power values for all nine conditions		Frequency bins significantly different from the control condition (p<0.05) are marked on the figure	Not shown
**3**	**d**	Wilcoxon signed rank test, two-sided	55	Cells from 8 rats	Full dataset shown	P = 0.0014	Z = 3.2003

4	**e**	Pearson's linear correlation	48	Gradient values from 4 cadavers	Box plots with whiskers denote medians, interquartile ranges and full ranges	P < 0.001	R = 0.5254
	**f**	One-way ANOVA with Bonferroni corrections	900	Gradient values from 5 cadavers		P = 0.99	F(8, 891) = 0.0667
	**g**	Paired t-test	60	Gradient values from 2 cadavers		P < 0.001 in all cases	t1(59) = -28.74;<br>t2(59) = -29.8515;<br>t3(59) = -22.541;<br>t4(59) = -21.5798;<br>t5(59) = -16.858;<br>t6(59) = -20.7634
5	**b**	Pearson's linear correlation	14 (skin),<br>81 (skull)	Stimulus intensity values from 6 (skin) and 10 (skull) cadavers	Raw data and fitted line	P < 0.001 in all cases	R(skull) = 0.9231;<br>R(skin) = 0.8593
	**c**	Pearson's linear correlation	29	Gradient values from 10 cadavers		P < 0.001	R = 0.56
	**d**	Pearson's linear correlation	16	Gradient values from 6 cadavers		P < 0.001	R = 0.8019
	**e**<br>skin-vs. skull	Paired t-test	36	Gradient values from 6 cadavers	Box plots with whiskers denote medians, interquartile ranges and full ranges	P < 0.001	t(35) = -9.7634
	**e**<br>skull-vs. brain	Paired t-test	60	Gradient values from 3 cadavers		P < 0.001	t(59) = -9.7461

**6**	**b**<br>left	Paired t-test with Bonferroni correction for multiple comparison	45	Trials from a single subject	Colormap of 500x6 medians (phases x intensities)	P = 1.811, 1.811, 0.08, <0.001, <0.001<br>for 1.5, 3, 4.5, 6, 7.5 mA,<br>all vs 0 mA	t(44) = 0.82, 0.92, 2.66, 5.07, 5.98<br>for 1.5, 3, 4.5, 6, 7.5 mA,<br>all vs 0 mA
	**b**<br>right	Paired t-test with Bonferroni correction for multiple comparison	45	Trials from a single subject	Colormap of 500x6 medians (phases x intensities)	P = 0.1, 1.65, 0.85, <0.001, <0.001<br>for 1.5, 3, 4.5, 6, 7.5 mA,<br>all vs 0 mA	t(44) = -2.56, 0.8, 1.5, 6.14, 6.71<br>for 1.5, 3, 4.5, 6, 7.5 mA,<br>all vs 0 mA
	**c**<br>left trough	Paired t-test with Bonferroni correction for multiple comparison	1025	Trials from 18 subjects	Box plots with whiskers denote medians, interquartile ranges and full ranges	P = 1.55, 3.89, 1.00, 0.006, <0.001<br>for 1.5, 3, 4.5, 6, 7.5 mA,<br>all vs 0 mA	t(1024) = 1.41, 0.45, -1.73, -3.60, -10.13<br>for 1.5, 3, 4.5, 6, 7.5 mA,<br>all vs 0 mA
	**c**<br>left peak	Paired t-test with Bonferroni correction for multiple comparison	1025	Trials from 18 subjects		P = 3.36, 0.22, 0.01, <0.001, <0.001<br>for 1.5, 3, 4.5, 6, 7.5 mA,<br>all vs 0 mA	t(1024) = -0.70, -2.41, -3.35, -5.92, -7.29<br>for 1.5, 3, 4.5, 6, 7.5 mA,<br>all vs 0 mA
	**c**<br>right peak	Paired t-test with Bonferroni correction for multiple comparison	1025	Trials from 18 subjects		P = 4.32, 2.60, 1.32, 0.58, <0.001<br>for 1.5, 3, 4.5, 6, 7.5 mA,<br>all vs 0 mA	t(1024) = -0.17, -0.98, -1.55, -2.00, -7.04<br>for 1.5, 3, 4.5, 6, 7.5 mA,<br>all vs 0 mA
	**c**<br>right trough	Paired t-test with Bonferroni correction for multiple comparison	1025	Trials from 18 subjects		P = 1.81, 0.07, 0.01, <0.001 , <0.001<br>for 1.5, 3, 4.5, 6, 7.5 mA,<br>all vs 0 mA	t(1024) = -1.27, -2.85, -3.35, -8.97, -10.23<br>for 1.5, 3, 4.5, 6, 7.5 mA,<br>all vs 0 mA

	**c**	Paired t-test with Bonferroni correction for multiple comparison	405, 408, 405, 404, 408 for 0, 2, 4.5, 7, 9 mA	Alpha power and gamma power values from a single subject		P(alpha) = 0.37, 0.42, <0.001, <0.001;<br>P(control) = 1.38, 1.38, 0.31, 0.62<br>for 2, 4.5, 7, 9 mA<br>all vs 0 mA;	Alpha: t(811) = -1.92, t(808) = -1.81, t(807) = 6.01, t(811) = 11.72;<br>Control: t(811) = -0.94, t(808) = -0.92, t(807) = 2.06, t(811) = 1.53;<br>for 2, 4.5, 7, 9 mA<br>all vs 0 mA;
	**d**	One-way ANOVA with Bonferroni correction	388x8, 391x8, 394x8, 393x8, 396x8, 394x8 for 'Pre0', 2, 4.5, 7, 9, 'Post0' mA intensities	Alpha power values from a single subject		P = 0.87, 0.85, 0.014, <0.001, <0.001, 0.14<br>for 'Pre0', 2, 4.5, 7, 9, 'Post0' mA intensities	F(7,3096) = 0.44;<br>F(7,3120) = 1.00;<br>F(7,3140) = 3.033;<br>F(7,3136) = 6.96;<br>F(7,3160) = 14.37;<br>F(7,3144) = 2.03;<br>for 'Pre0', 2, 4.5, 7, 9, 'Post0' mA intensities
**7**	**d**	Post-hoc paired t-test with Bonferroni correction for multiple comparison	388x8, 391x8, 394x8, 393x8, 396x8, 394x8 for 'Pre0', 2, 4.5, 7, 9, 'Post0' mA intensities	Alpha power values from a single subject	Error bars are mean ± SEM	P(Pre0) = 5.25, 5.69, 6.05, 5.89, 5.81, 1.77, 6.33, 6.67;<br>P(2 mA) = 6.03, 1.37, 5.5, 4.96, 6.67, 5.86, 6.16, 1.93;<br>P(4.5 mA) = 1.03, 0.005, 4.59, 2.42, 0.57, 2.56, 5.86, 5.69;<br>P(7 mA) = 5.8, 0.002, 0.53, 6.16, 0.78, 0.57, 0.005, 2.98;<br>P(9 mA) = 3.11, <0.001, <0.001, 5.06, 0.82, 6.10, 3.87, 1.86;<br>P(Post0) = 6.33, 5.52, 2.01, 0.76, 5.25, 6.10, 1.19, 1.48<br>for 45, 90, 135, 180, 225, 270, 360° each	T(387) = -1.29, -1.21, -0.69, 1.06, 1.03, -0.03, -0.73, -0.43 (‘Pre0’);<br>T(390) = 0.40, 2.07, 0.35, 1.35, 0.430, 0.65, -0.90, -1.88 (2 mA);<br>T(393) = 2.21, 3.90, -0.29, -1.75, -2.48, 0.14, -0.90, 1.20 (4.5 mA);<br>T(392) = -1.02, 4.09, 2.51, 0.91, 2.34, -2.47, -3.91, -1.63 (7 mA);<br>T(395) = -0.18, 7.56, 7.26, 0.30, 2.30, 1.09, -0.28, -1.91 (9 mA);<br>T(393) = -0.75, 1.18, 1.85, 2.35, 1.30, -1.08, -2.14, -2.02 (‘Post0’)<br>for 45, 90, 135, 180, 225, 270, 360° each
	**f**	Two sample Kolmogorov-Smirnov test	16, 10, 8, 18, 10, 12	Modulation vector lengths for '0pre', 2, 4.5, 7, 9 and '0post' intensities from a single subject		P=0.98, 0.041, <0.001, 0.019, 0.17<br>for 2, 4.5, 7, 9 and '0 Post0' intensities<br>all vs. 'pre0'	D = 0.17, 0.56, 0.81, 0.57, 0.39<br>for 2, 4.5, 7, 9 and 'post0' intensities<br>all vs. 'pre0'
	**g**	Paired t-test	23	Trials from a single subject		P = 0.96, 0.79, 0.44, 0.44 , 0.74, 0.11<br>for 0, 1.5, 3, 4.5, 6, 7.5 mA	t(22) = 0.044, -0.26, 0.78, 0.77, -0.32, -1.63<br>for 0, 1.5, 3, 4.5, 6, 7.5 mA

**8**	**b**<br>left	One sample t-test with Bonferroni correction for multiple comparison	809	Power difference values for all conditions	Full dataset & error bars are mean ± SEM	P = 0.36, <0.005, 1.68, <0.005, 0.41<br>for the five L-R conditions and<br>P = 1.06, <0.005 1.52, 1.29, 0.39<br>for the five R-L conditions	t(807) = 2.09, 16.47, 0.45, 6.47, 1.85<br>for the five L-R conditions and<br>t(807) = -1.56, 13.21, -1.02, 0.33, 1.95<br>for the five R-L conditions
	**b**<br>right	One sample t-test with Bonferroni correction for multiple comparison	809	Power difference values for all conditions		P = 2.22, <0.005, 2.22, 0.16, 1.68<br>for the five L-R conditions and<br>P = 1.45, <0.005, 1.50, 1.45, 1.14<br>for the five R-L conditions	t(807) = -0.57, 12.00, 0.59, -2.43, 1.38<br>for the five L-R conditions and<br>t(807) = -1.33, 18.17, 1.14, -1.32, 1.59<br>for the five R-L conditions
	**c**<br>left	Mann and Whitney U test with Bonferroni correction	125, 144, 117, 126, 127, 148 and 211	x 500 spectral power values for the seven consecutive conditions	Error bars are mean ± SEM	Frequency bins significantly different from open-eye conditions (p < 0.05) are marked on the figure	Not shown
	**c**<br>right	Mann and Whitney U test with Bonferroni correction	125, 144, 117, 126, 127, 148 and 211	x 500 spectral power values for the seven consecutive conditions		Frequency bins significantly different from open-eye conditions (p < 0.05) are marked on the figure	Not shown
**Supp 1**	**c**	Pearson's linear correlation	3053, 3092, 3098 for 0, 2 and 9 ma stimulus intensities	Instantaneous alpha frequency values from a single subject	Full distributions are shown in dot plots	P = 0.89, 0.57 and 0.82<br>for 0, 2 and 9 mA stimulus intensities	R = -0.0024, 0.01 and -0.004<br>for 0, 2 and 9 mA stimulus intensities
**Supp 4**	**c**	Mann and Whitney U test	10	Gradient values from 2 cadavers	Error bars are mean ± SEM	P = 0.1212	Z = 1.5497
**Supp 8**	**i**	Pearson's linear correlation	128	Gradient values in cortex from 8 cadavers	Median and the interquartile range	P = 0.00833	R = -0.232
